# Supplementary material for: Neural Progenitors Adopt Specific Identities by Directly Repressing All Alternative Progenitor Transcriptional Programs
Source: Dev Cell. 2016 Mar 21;36(6):639–53. doi: 10.1016/j.devcel.2016.02.013 (PMC4819439; doi:10.1016/j.devcel.2016.02.013)
Supplement: Document S1. Supplemental Experimental Procedures and Figures S1–S5 [file mmc1.pdf]

Developmental Cell, Volume *36*

Supplemental Information

Neural Progenitors Adopt Specific Identities by Directly Repressing All Alternative Progenitor Transcriptional Programs

Eva Kutejova, Noriaki Sasai, Ankita Shah, Mina Gouti, and James Briscoe

Inventory of Supplemental Materials

Supplemental Figures and Legends

Figure S1. Gene ontology, binding motifs. Related to Figure 1.

Figure S2. Inducible ES cell lines. Related to Figures 2, 3, 4 and 5.

Figure S3. Identification of genes specific for definitive FP – "late" FP genes. Related to Figure 2 and Table S1, sheet S3.

Figure S4. Expression kinetics of genes induced by Shh. Shh-regulated genes associate with activator and repressor peaks. Related to Figure 6 and Table S1, sheet S4.

Figure S5. Activity of individual ventral CREs correlates with direct positive input of Sox2/3 and Gli1 and direct negative inputs of Nkx2.2, Olig2 and Nkx6.1. Related to Figures 5 and 6.

Supplemental Tables (available as separate files)

Table S1. Genes, their expression and classification. Related to Figures 1D, 2B, 2F, 3B, 3F, 3G, S3B-C and S4A.

Table S2. Lists of RNA and ChIP-seq samples, accession numbers. Related to Figures 1, 2, 3, 4, 5, 6, S1, S3, S4 and S5.

Table S3. RNA-seq: read counts. Related to Figures 1, 2, 3, 4, 5, 6, S1, S3 and S4.

Table S4. ChIP-seq: peaks. Related to Figures 1, 2, 3, 5, 6, S1, S4 and S5.

Table S5. Gene lists and samples used to generate heatmaps and boxplots. Related to Figures 1, 2, 3, 4, 5, 6, S1, S3 and S4.

Supplemental Experimental Procedures

Antibodies for immunohistochemistry.

Production of recombinant C25II-Shh protein.

Production of anti-Nkx2.2 and anti-Nkx6.1 antibodies.

ChIP-seq analysis.

RNA-seq analysis. Gene lists. Heatmaps. Boxplots.

Supplemental References

Supplemental Figures and Legends

Figure S1

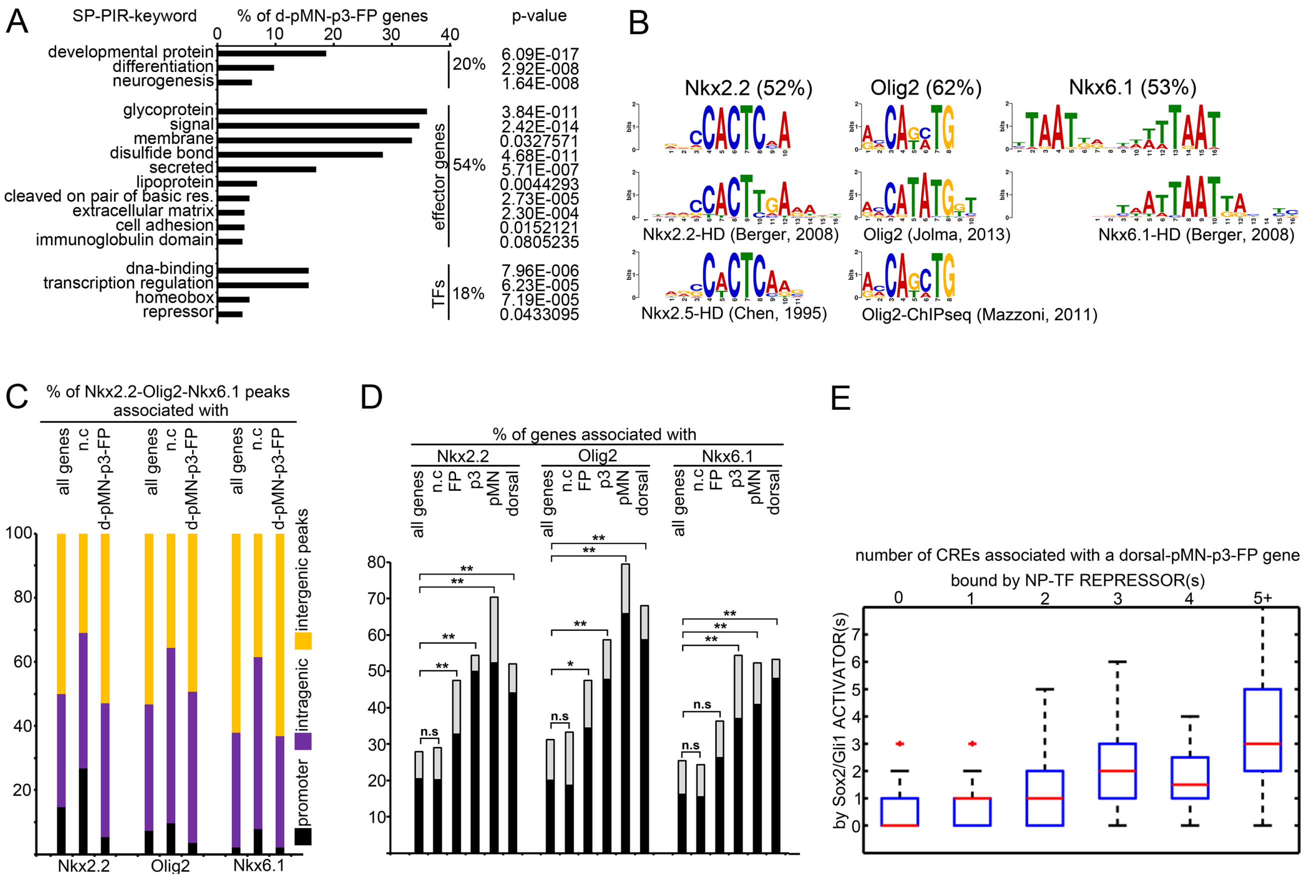

Figure S1. Gene ontology, binding motifs. Related to Figure 1.

A. Gene ontology analysis indicates that genes differentially expressed in FP-p3-pMN-dorsal (Fig. 1D) are enriched in TFs (18%) and membrane-associated/secreted molecules (54%) compared to all genes, p<0.1. The analysis was performed using DAVID (Huang et al., 2009) and is based on Swiss-Prot (SP) and Protein Information Resource (PIR) Keywords (SP_PIR_KEYWORD).

B. De novo motifs searches from regions bound by Nkx2.2, Olig2, Nkx6.1 recovered the corresponding *in vitro* defined motifs (Berger et al., 2008; Chen and Schwartz, 1995; Jolma et al., 2013) and motifs recovered from other ChIP-seq datasets (Olig2) (Mazzoni et al., 2011).

C. Nkx2.2, Olig2 and Nkx6.1 bind to intragenic and intergenic regions rather than promoters. Based on their genomic position with respect to the RefSeq transcripts, peaks were first classified into promoter (TSS-3kb to TSS, black), intragenic (TSS to TES, yellow, more than 70% intronic) and intergenic (other, purple) peaks and then associated with the closest genes. All three TFs were depleted from the promoter regions, especially from the promoters of the regulated genes - genes differentially expressed in dorsal-pMN-p3-FP (Fig. 1D), (black).

D. The proportion of dorsal-pMN-p3-FP genes associated with Nkx2.2-Olig2-Nkx6.1 further increases when two closest genes on each side of the peak are analysed. Compare black bars (one closest gene, Fig. 1E) with grey bars (two closest genes). The enrichment compared to all genes is highly significant, especially when the promoter peaks of Nkx2.2 (that do not seem to confer regulatory properties, Fig. S1C) are excluded (shown here for two closest genes). ** $p(X^2)<0.001$, * $p(X^2)<0.025$.

E. Dorsal-pMN-p3-FP genes that lack a CRE bound by NP-TFs in their immediate vicinity also tend to lack binding of activator TFs Sox2 and Gli1. The box plot indicates the number of Sox2 and/or Gli1 bound CREs compared to number of NP-TF bound CREs for each regulated gene. This agrees with the observation that the regulatory modules associated with a gene or a set of genes tend to cluster. D and E suggest that the regulated genes not harbouring NP-TF bound CREs in their immediate vicinity are likely to be controlled by CREs separated by one or more genes.

Figure S2

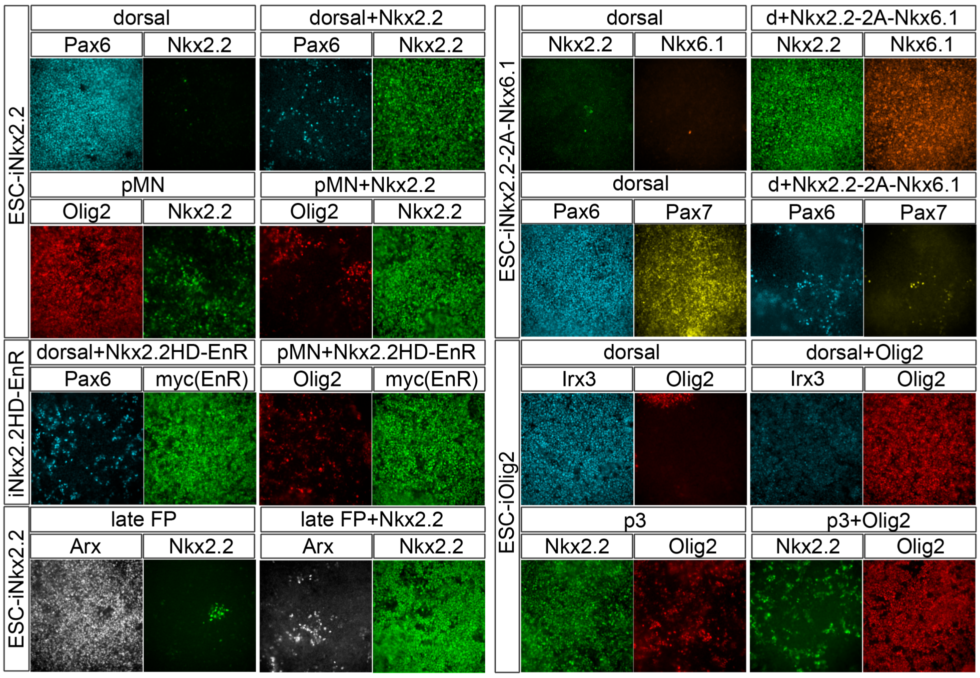

Figure S2. Inducible ES cell lines. Related to Figures 2, 3, 4 and 5.

Expression of Nkx2.2 (ESC-iNkx2.2 cell line) in dorsal, pMN and FP cells leads to the downregulation of the corresponding TFs Pax6, Olig2 and Arx. Similarly, overexpression of Nkx2.2HD-EnR using ESC-iNkx2.2HD-EnR cell line in dorsal and pMN abolishes expression of Pax6 and Olig2. Expression of Nkx2.2 and Nkx6.1 (ESC-iNkx2.2-2A-Nkx6.1) in dorsal cells downregulates Pax6 and Pax7. Expression of Olig2 (iNPC-Olig2) in dorsal and p3 cells represses Irx3 and Nkx2.2 respectively. Expression of marker genes in all conditions was analysed by immunofluorescence at day 5 (36h) except for the late FP, where the pictures were taken at day 6 (60h).

Figure S3

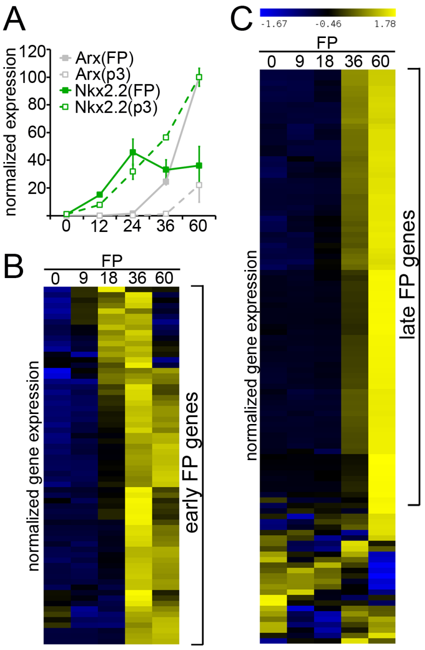

Figure S3. Identification of genes specific for definitive FP – "late" FP genes. Related to Figure 2 and Table S1, sheet S3.

A. The FP marker Arx is induced between 36 and 60h, at the time Nkx2.2 expression is downregulated.

B, C. Classification of FP genes. Genes expressed higher in iNPC-FP compared to iNPC-p3 at 36 and/or 60h were classified according to their time of induction as "early" FP genes, induced to maximum levels at 18 or 36h (B), or as "late" FP genes, induced to maximum levels at 60h (C).

Figure S4

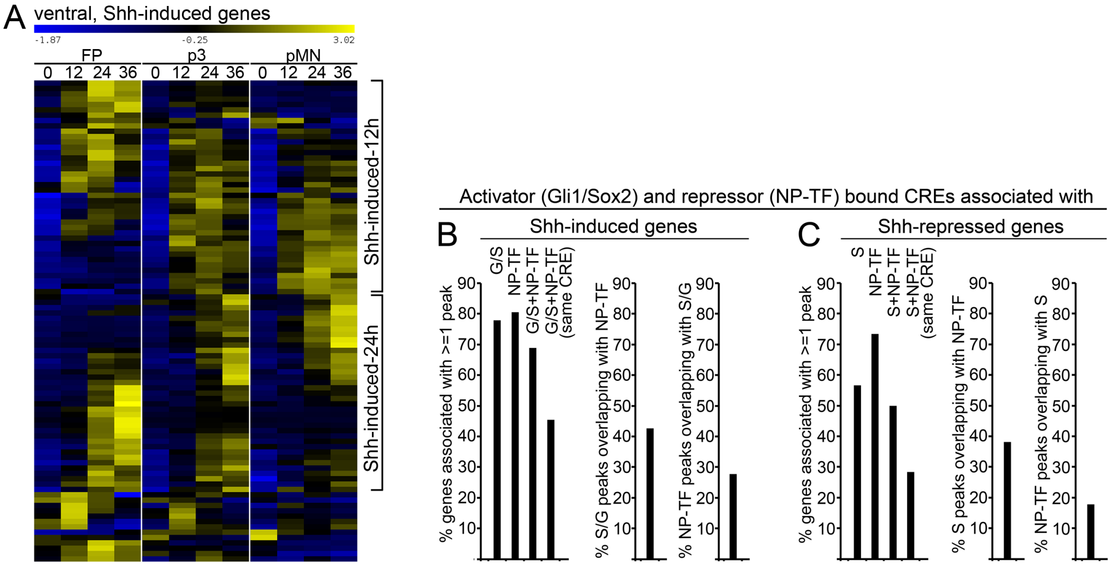

Figure S4. Expression kinetics of genes induced by Shh. Shh-regulated genes associate with activator and repressor peaks. Related to Figure 6 and Table S1, sheet S4.

A. Genes induced at 12h in FP, p3 or pMN were classified 'ventral-induced by Shh at 12h'. Genes induced at 24h in FP, p3 or pMN were classified 'ventral-induced by Shh at 24h'.

B-C. Most Shh-regulated genes associate with both activator and repressor peaks. Only a fraction of gene-associated CREs are co-occupied by activator and repressor TFs.

B. 78/81% of Shh-induced genes are associated with an activator (G/S, Gli1 and/or Sox2) or a repressor (NP-TF, Nkx2.2 or Olig2 or Nkx6.1), respectively. 88% of Sox2 and/or Gli1 associated genes bind a repressor as well (58% of those have at least one shared S/G+NP-TF CRE). 43% of activator bound peaks next to Shh-induced genes are bound by a repressor. 28% of repressor bound peaks are bound by an activator.

C. 57% of Shh-repressed genes are associated with an activator (S, Sox2), 73% with a repressor (NP-TF, Nkx2.2 or Olig2 or Nkx6.1). 88% of Sox2 associated genes bind a repressor as well (50% of those have at least one shared S+NP-TF CRE). 38% of activator bound peaks next to Shh-repressed genes are bound by a repressor. 18% of repressor bound peaks next to Shh-repressed genes is associated with an activator peak.

Figure S5

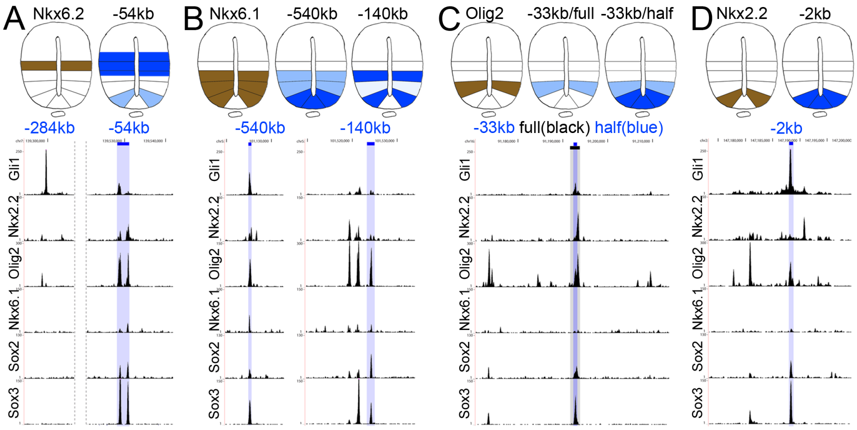

Figure S5. Activity of individual ventral CREs correlates with direct positive input of Sox2/3 and Gli1 and direct negative inputs of Nkx2.2, Olig2 and Nkx6.1. Related to Figures 5 and 6.

The schematics summarize dorsal-ventral restricted activities in the neural tube of the indicated CREs (blue, darker shades indicate higher expression) and compare them to domain-specific expression of the associated genes (brown). The tracks correspond to binding of Nkx2.2, Nkx6.1 and Sox2 in p3 and Olig2 in pMN cells. Gli1 tracks were generated by remapping the raw reads from (Peterson et al., 2012). Sox3 tracks were remapped from (Bergsland et al., 2011).

A. Nkx6.2(-54kb) double CRE which is strongly expressed in p2/p1-p0 domains and weakly in the p3 domain, is bound by Gli1, Sox TFs and Olig2 in pMN and by Nkx2.2 in p3 domains. CRE activity adapted from (Peterson et al., 2012).

B. Nkx6.1(-540kb) CRE which is strongly expressed in FP and p3 and weakly in pMN and p2, receives inputs from Gli1 and Sox TFs as well as from Olig2 and Nkx6.1. CRE activity adapted from (Peterson et al., 2012). Nkx6.1(-140kb) CRE expressed in p2 and p3 domains, receives positive inputs from Sox TFs and negative input from Olig2 in the pMN domain. CRE activity adapted from (Peterson et al., 2012).

C. The complete Olig2(-33kb) CRE is bound by Sox/Gli1 and by Olig2 and Nkx2.2. Its activity is detected in pMN and it is excluded from p3. The full CRE contains one Gli1 site, two Olig2 sites and an Nkx2.2 binding site. The truncated version of the same CRE, Olig2(-33kb/half), which lacks the Nkx2.2 element but retains the Sox, Gli and one of the Olig2 binding sites is active in the p3 domain consistent with a role for Nkx2.2 binding in the repression of this element in p3 domain the pMN domain. The Olig2 binding correlates with ability of Olig2 to repress its own expression (Fig. 5F). Full CRE activity adapted from (Oosterveen et al., 2012). Half CRE activity adopted from (Peterson et al., 2012).

D. p3-restricted activity of Nkx2.2(-2kb) CRE is consistent with the activatory inputs of Gli1/Sox binding and direct repression by Olig2 and more dorsal TFs (e.g Pax6, Irx3). The CRE activity was adapted from (Oosterveen et al., 2012; Peterson et al., 2012).

Supplemental Tables

Table S1. Genes, their expression and classification related to Figures 1D, 2B, 2F, 3B, 3F, 3G, S3B-C and S4A.

Sheet 1D: list of gene IDs, gene names, gene classification, SP-PIR-keywords, *in vivo* expression patterns, normalized expression values and p-values corresponding to the heatmap in Fig. 1D (dorsal-pMN-p3-FP progenitor genes). FP>p3>>pMN genes (Fig. 5B) and pMN>d genes (Fig. 5F) are indicated. The sheet 1D also contains the list of references corresponding to the *in vivo* gene expression patterns (bottom of the page).

Sheet 2B: list of gene IDs, gene names, gene classification, normalized expression values and p-values corresponding to the heatmap in Fig. 2B (pMN genes and pMN genes repressed by Nkx2.2).

Sheet 2F: list of gene IDs, gene names, SP-PIR-keywords, *in vivo* expression patterns, gene classification, normalized expression values and p-values corresponding to the heatmap in Fig. 2F (late FP genes and FP genes repressed by Nkx2.2). Sheet 2F also contains the list of references corresponding to the *in vivo* gene expression patterns (bottom of the page).

Sheet 3B: list of gene IDs, gene names, gene classification, normalized expression values and p-values corresponding to the heatmap in Fig. 3B (dorsal genes repressed in p3 by Nkx2.2 and/or Nkx6.1).

Sheet 3F: list of gene IDs, gene names, gene classification, normalized expression values and p-values corresponding to the heatmap in Fig. 3F (dorsal genes repressed in p3 and/or pMN).

Sheet 3G: list of gene IDs, gene names, gene classification, normalized expression values and p-values corresponding to the heatmap in Fig. 3G (dorsal genes repressed in pMN by Olig2).

Sheet S3: list of gene IDs, gene names, gene classification, normalized expression values and p-values corresponding to the heatmaps in Fig. S3B-C (early and late FP genes).

Sheet S4: list of gene IDs, gene names, gene classification, normalized expression values and p-values corresponding to the heatmaps in Fig. S4A (ventral genes activated by Shh at 12h and 24h).

Table S2. Lists of RNA and ChIP-seq samples, accession numbers. Related to Figures 1, 2, 3, 4, 5, 6, S1, S3, S4 and S5.

Sheet RNA-seq: list of conditions, corresponding RNA-seq samples and their ENA accession numbers.

Sheet ChIP-seq: list of ChIP-seq samples and their ENA accession numbers.

Table S3. RNA-seq: read counts. Related to Figures 1, 2, 3, 4, 5, 6, S1, S3 and S4.

Columns 1-140: Read counts - RNA-seq samples.

Columns 142-281: Read counts normalized - RNA-seq samples.

Table S4. ChIP-seq: peaks. Related to Figures 1, 2, 3, 5, 6, S1, S4 and S5.

Sheet Nkx2.2: position and read coverage of Nkx2.2 peaks (p3 cells).

Sheet Olig2: position and read coverage of Olig2 peaks (pMN cells).

Sheet Nkx6.1: position and read coverage of Nkx6.1 peaks (p3 cells).

Sheet Gli1: position and read coverage of Gli1 peaks (Peterson et al., 2012).

Sheet Sox2-d: position and read coverage of Sox2 peaks (dorsal cells).

Sheet Sox2-p3: position and read coverage of Sox2 peaks (p3 cells).

Sheet Genes-Peaks: List of RefSeq genes and associated peaks.

Table S5. Gene lists and samples used to generate heatmaps and boxplots. Related to Figures 1, 2, 3, 4, 5, 6, S1, S3 and S4.

The table contains multiple sheets, each sheet corresponds to the indicated figure panel.

Supplemental Experimental Procedures

Production of anti-Nkx2.2 and anti-Nkx6.1 antibodies

The N-terminal fragment of mouse Nkx2.2 (amino acids 26 to 121) was expressed as a His-tag fusion in E. *coli*. The C-terminal fragment of mouse Nkx6.1 (amino acids 300 to 365) was expressed as a GST-3C-Nkx6.1 fusion in E. coli. The GST was removed using HRV 3C protease (Millipore). Rabbits were immunized with purified proteins by Harlan, UK under licence PPL60/3418 following their predefined 77 day immunization schedule. The project was initiated following standard Ethical Review Process of Harlan Laboratories. The antibodies were affinity purified from the sera by coupling the immunogens to NHS-activated Sepharose (GE Healthcare).

Production of recombinant C25II-Shh protein

Mouse N-terminal fragment of Shh (amino acids 25-198, where the N-terminal cysteine 25 was replaced by two isoleucines) (Pepinsky et al., 1998) was cloned into pET22b downstream of a His-tag followed by enterokinase cleavage site, expressed and purified as described previously for unmodified human N-terminal Shh fragment (Williams et al., 1999).

Antibodies for immunohistochemistry

Following antibodies were used to stain chick sections and/or *in vitro* differentiated neural progenitor cells: Arx (rabbit, gift from J. Chelly (Poirier et al., 2004)), Dbx1 (rabbit, gift from T. Jessell (Vallstedt et al., 2001)), Foxa2 (rabbit, Seven Hills WRAB-1200 or goat, Santa Cruz sc-6554X), Irx3 (gift from T. Jessell (Novitch et al., 2001)), Nkx2.2 (mouse, DSHB 74.5A5 or rabbit, this manuscript), Olig2 (rabbit, Millipore AB9610), Nkx6.1 (mouse, F55A10 and F64A6B4, gift from O. Madsen, BCBC Antibody Core), Pax6 (rabbit, Millipore AB2237), Pax7 (DSHB).

ChIP-seq analysis

36-bp single-end reads were aligned to GRCm38 genome assembly using Bowtie (-v 0 -m 1 –best –strata) (Langmead et al., 2009). MACS14 (Zhang et al., 2008) was used to call peaks (-m 10, 200 -bw 100 –nolambda). Ten thousand peaks with the highest -10*log10(pvalue) from each dataset were selected for further analysis. Peak Splitter and the top four thousand peaks from each dataset were used to call subpeaks. Peak lists were combined and the number of reads per peak recalculated covering 200pb (250bp for Gli1) on each side of the peak summit, allowing no duplicate reads to map to the same peak. Peaks on chromosomes Y and M and peaks overlapping with the following regions: chr2:98,666,255-98,667,005, chr9:3,000,070-3,027,155, chr11:3,086,238-3,224,949, chr14:19,415,511-19,419,916, chr17:39,842,761-39,849,213 were eliminated from further analysis. These regions contain repetitive sequences where reads accumulate in a non-specific manner in all datasets analysed. Distribution of peak heights was approximated using log-normal distribution fit and peaks with height corresponding to cumulative p-value above 0.67 (Nkx2.2, Olig2, Nkx6.1, Sox2) or 0.9 (Gli1) were used for the analysis. This corresponds to 2644 Nkx2.2 peaks (minimum 62 reads), 3080 Olig2 peaks (minimum 188 reads), 2479 Nkx6.1 peaks (minimum 61 reads) and 860 Gli1 peaks (minimum 70 reads). Sox2 ChIP-seq was performed from p3 (2200 peaks, minimum 36 reads) and dorsal progenitors (2350 peaks, minimum 37 reads). For Fig. 6C and Fig. S4B-C, the highest peaks called from the two datasets which show 65% overlap were pooled and associated with the closest genes. For full peak lists including heights, see Table S4. Peaks were associated with the closest genes (-1, +1, within 1MB) using CisGenome (Ji et al., 2008) and gene reference table containing GRCm38 RefSeq genes (Table S4, sheet Genes-Peaks). For numbers of peaks associated with each gene, also see Table S4, sheet Genes-Peaks. De-novo motif search was performed using Homer (Heinz et al., 2010) and the sequence covering 100bp on each side from the peak summit. TomTom (Gupta et al., 2007) was used to search for similar motifs in known datasets.

RNA-seq analysis. Gene lists. Heatmaps. Boxplots.

The paired-end reads were aligned to GRCm38 genome using Tophat (default parameters) (Trapnell et al., 2009). The number of reads corresponding to all features in Ensembl Genes 77 GTF table were counted using HTSeq (Anders et al., 2015). See Table S3, columns 1-140 for raw read counts. The pairwise differential expression analysis was performed using DESeq (Anders and Huber, 2010) using raw read counts. Genes with very low counts across the whole dataset were removed prior to analysis (Table S3, column 141, “removed”). Otherwise, samples were normalized to the total number of reads (Table S3, columns 142-181). All cut-offs refer to the normalized values. MEV 4.0 (www.tm4.org) (Saeed et al., 2003) was used for hierarchical clustering and to generate expression heatmaps. “padj” refers to adjusted p-value (n-binomial) as defined by DESeq. “n.c” genes represent 600 RefSeq genes with minimal fold change across the RNAseq data set, the “n.c” genes are highlighted in (Table S4, sheet Genes-Peaks).

Bedtools (Quinlan and Hall, 2010), Samtools (Li et al., 2009) were used to interrogate, process and combine the ChIP-seq and RNA-seq datasets.
Samples and conditions used to generate heatmaps and boxplot panels are described below. For details of gene lists, see Table S5.

Fig. 1D – Table S5, sheet Fig1D

To classify genes differentially expressed in dorsal(30RA), p3, pMN and FP, gene lists 1D-1 (36h-dorsal(30RA) higher than 36h-p3), gene lists 1D-2 (36h-dorsal(300RA) higher than 36h-pMN), 1D-3 minus 1D-6 (36h-p3 higher than 36h-pMN), 1D-4 minus 1D-7 (pMN higher than p3) and 1D-4 (early FP higher than p3) were combined. To generate the heatmap, the average expression values from the replicate samples (1D-8) corresponding to 36h-dorsal(30RA), 36h-p3, 36h-pMN, 36h-FP conditions were normalized across the four conditions (z-scores). The genes were classified according their normalized expression values into Fig. 1D-"dorsal", Fig. 1D-"p3", Fig. 1D-"pMN", Fig. 1D-"FP" genes.

"Dorsal" genes are expressed higher in 36h-dorsal(30RA) than 36h-p3 and pMN and they are expressed in 36h-dorsal(30RA) or 36h-dorsal(30RA)/FP. "pMN" genes are expressed higher in 36h-pMN than 36h-p3 and expressed in 36h-pMN, 36h-pMN/dorsal or 36h-pMN/FP. "p3" genes are expressed higher in 36h-p3 than 36h-pMN and 36h-dorsal, they are expressed in 36h-p3 or 36h-p3/FP. For full list details, including p-values and the classification of the genes from the merged list corresponding to Fig. 1D, see Table S1, sheet 1D.

Fig. 2B – Table S5, sheet Fig2B

To identify pMN genes repressed by Nkx2.2, the gene list defined in Fig. 1D as "pMN" (expressed higher in 36h-pMN than 36h-p3 and expressed in 36h-pMN, 36h-FP and 36h-pMN or 36h-dorsal and 36h-pMN) was used.

To generate the heatmap, the average expression values from the replicate samples (2B-1) corresponding to 0h-pMN, 12h-pMN, 24h-pMN, 36h-pMN, 24h-pMN+Nkx2.2, 36h-pMN+Nkx2.2 samples were normalized across all conditions (z-scores). Genes were classified according to their time of induction into early, intermediate and late. padj was calculated from 2B-2-padj comparing expression values in 36h-pMN and 36h-pMN+Nkx2.2. pMN genes repressed by Nkx2.2 with padj<0.05 were classified "repressed by Nkx2.2". For full list details, including p-values and classification, see Table S1, sheet 2B.

Fig. 2F -Table S5, sheet Fig2F

To identify late FP genes repressed by Nkx2.2, the gene list defined as "late" FP genes in Fig. S3C (expressed higher in 60h-FP than 60h-p3 and induced to maximum levels at 60h) was used. To generate the heatmap, the average expression values from the replicate samples (2F-1) corresponding to 0h-FP, 9h-FP, 18h-FP, 36h-FP, 60h-FP, 18h-FP+Nkx2.2, 36h-FP+Nkx2.2, 60h-FP+Nkx2.2 were normalized across all conditions (z-scores). padj was calculated from 2F-2-padj60 comparing expression values in 60h-FP and 60h-FP+Nkx2.2. For full list details, including p-values, see Table S1, sheet 2F.

Fig. 3B – Table S5, sheet Fig3B

To identify dorsal genes repressed by Nkx2.2, Nkx6.1 and/or Nkx2.2+Nkx6.1, defined in Fig. 1D as "dorsal" (expressed higher in 36h-dorsal(30RA) than 36h-p3 and 36h-p3) was used. To generate the heatmap, the average expression values from the replicate samples corresponding to 36h-dorsal(30RA), 36h-p3, 36h-dorsal+Nkx2.2, 36h-dorsal+Nkx6.1, 36h-dorsal+Nkx2.2+Nkx6.1 conditions from the replicate samples (3B-1) were normalized across all conditions (z-scores). Genes were classified into "downregulated by Nkx2.2", "downregulated by Nkx6.1" and "downregulated by Nkx2.2 and Nkx6.1" based on the level of repression compared to 36h-dorsal(30RA). For full list details, including p-values and classification, see Table S1, sheet 3B.

Fig. 3F - Table S5, sheet Fig3F

To identify dorsal genes repressed in p3 and/or pMN, two lists were combined: 3F-1 (higher in 36h-dorsal(30RA) than 36h-p3) and 3F-2 (higher in 36h-dorsal(300RA) than 36h-pMN). Average values from the replicate samples in 36h-p3 and 36h-pMN were expressed as percent of 36h-dorsal(30RA) and 36h-dorsal(300RA) average values respectively, 36h-dorsal(30RA) and 36h-dorsal(300RA) average values were set to 100. To generate the heatmap, percent values corresponding to 36h-dorsal, 36h-p3 and 36h-pMN were then normalised across the three conditions. Genes were classified into "dorsal repressed in pMN", "dorsal repressed in p3" and "dorsal repressed in p3 and pMN" based on the level of repression compared to 36h-dorsal. For full list details, including classification, see Table S1, sheet 3F.

Fig. 3G - Table S5, sheet Fig3G

To identify 36-dorsal(300RA) genes repressed by Olig2, the gene list including genes expressed higher in dorsal(300RA) than pMN, defined as "repressed in pMN" in Fig. 3F was used. To generate the heatmap, the average expression values from the replicate samples (3G-1) corresponding to 36h-dorsal(300RA), 36h-pMN, 36h-dorsal(300RA)+Olig2 conditions were normalized across all conditions (z-scores). Genes were classified into "downregulated by Olig2" based on the level of repression compared to 36h-dorsal(300RA). For full details, including p-values and classification, see Table S1, sheet 3G.

Fig. 4B - Table S5, sheet Fig4B

To generate the boxplots corresponding to 36-dorsal and 36-pMN conditions when Nkx2.2, Nkx2.2 and Nkx6.1 or Nkx2.2HD-EnR were induced, "dorsal" genes (higher in 36h-dorsal(30RA) than 36h-p3 and pMN, expressed in 36h-dorsal(30RA) or 36h-dorsal(30RA)/FP), "pMN" genes (higher in 36h-pMN than 36h-p3, expressed in 36h-pMN, 36h-pMN/dorsal or 36h-pMN/FP) and "p3" (higher in 36h-p3 than 36h-pMN and 36h-dorsal, expressed in 36h-p3 or 36h-p3/FP) gene lists from Fig. 1D were used.

For the boxplot of 'dorsal' gene behaviour, the median expression values from the replicate samples (4B-1-d) corresponding to 36h-dorsal(30RA), 36h-p3, 36h-dorsal(30RA)+Nkx2.2, 36h-dorsal(30RA)+Nkx2.2+Nkx6.1 and 36h-dorsal(30RA)+Nkx2.2HD-EnR conditions, were normalized across all conditions (z-scores).

For the boxplot of 'pMN' gene behaviour, the median expression values from the replicate samples (4B-2-pMN) corresponding to 36h-pMN, 36h-p3, 36h-pMN+Nkx2.2 and 36h-pMN+Nkx2.2HD-EnR conditions, were normalized across all conditions (z-scores).

For 'p3' gene behaviour, the median expression values from the replicate samples (4B-3-p3) corresponding to 36h-dorsal(30RA), 36h-p3, 36h-dorsal(30RA)+Nkx2.2, 36h-dorsal(30RA)+Nkx2.2+Nkx6.1, 36h-dorsal(30RA)+Nkx2.2HD-EnR, 36h-pMN, 36h-pMN+Nkx2.2 and 36h-pMN+Nkx2.2HD-EnR conditions were normalized across all conditions (z-scores). Nkx2.2 read count in 36h-dorsal(30RA)+Nkx2.2, 36h-dorsal(30RA)+Nkx2.2+Nkx6.1 and 36h-dorsal(30RA)+Nkx2.2HD-EnR conditions was set to 600.

Fig. 4C - Table S5, sheet Fig4C

To generate the cross-correlation plots of dorsal/p3 and pMN/p3 gene behaviour when Nkx2.2, Nkx6.1, Nkx2.2 and Nkx6.1 or Nkx2.2HD-EnR were overexpressed, we used the same gene lists and same samples that were analysed in Fig. 4B.

For the cross-correlation of 'd-p3 genes', the median expression values from the replicate samples (4C-1-d-p3) corresponding to 36h-dorsal(30RA), 36h-p3, 36h-dorsal(30RA)+Nkx2.2, 36h-dorsal(30RA)+Nkx6.1, 36h-dorsal(30RA)+Nkx2.2+Nkx6.1, 36h-dorsal(30RA)+Nkx2.2HD-EnR conditions, were normalized across all conditions (z-scores).

For the cross-correlation of 'pMN-p3 genes', the median expression values from the replicate samples (4C-2-pMN-p3) corresponding to 36h-pMN, 36h-p3, 36h-pMN+Nkx2.2 and 36h-pMN+Nkx2.2HD-EnR conditions, were normalized across all conditions (z-scores). Nkx2.2 read count in 36h-dorsal(30RA)+Nkx2.2, 36h-dorsal(30RA)+Nkx2.2+Nkx6.1 and 36h-dorsal(30RA)+Nkx2.2HD-EnR conditions was set to 600.

Fig. 5B - Table S5, sheet Fig5B

To generate the boxplots corresponding to 36-FP, 36-FP+Nkx2,2, 36-p3, 36pMN+Nkx2.2, “FP>p3>>pMN” genes (“p3” genes expressed higher in 36h-FP than 36h-p3) from Fig. 5B, green arrow, were used. The average expression values from the replicate samples (5B-1) were normalized across all conditions (z-scores).

Fig. 5C - Table S5, sheet Fig5C

To identify “FP>p3>>pMN” genes downregulated by Nkx2.2, replicate samples corresponding to 36-FP and 36-FP+Nkx2.2 (list 5C-1) were compared.

Fig. 5F - Table S5, sheet Fig5F

To generate the boxplots corresponding to 24-dorsal, 24-dorsal+Olig2, 24-ventral, 24-ventral+Olig2, “pMN-only” genes (“pMN” genes expressed higher in 36h-pMN than 36h-p3 and 36h-dorsal) from Fig. 5F, red arrow, were used. The average expression values from the replicate samples (5F-1) were normalized across all conditions (z-scores). Olig2 values were adjusted according to qPCR data from the same RNA samples, using primers that do not amplify Olig2 cDNA.

Fig. 5G - Table S5, sheet Fig5G

To generate the boxplots corresponding to 24-dorsal, 24-ventral, 24-ventral+Olig2, “ventral” genes expressed in both p3 and pMN (list 5G-1) were used. The average expression values from the replicate samples (5G-1) were normalized across all conditions (z-scores).

Fig. 5H - Table S5, sheet Fig5H

To identify pMN-specific and ventral genes downregulated by Olig2, replicate samples corresponding to 24-p3 and 24-p3+Olig2 were compared (list 5H-1).

Fig. 6B - Table S5, sheet Fig6B

Ventral genes induced by Shh at 12h or 24h were defined as described in Fig. S4A.

To define dorsal genes repressed by Shh at 24h, two gene lists were combined: 6B-3-Shh-repressed (higher in 24h-dorsal(30RA) than 24h-p3) and 6B-4-Shh-repressed (higher in 24h-dorsal(300RA) than 24h-pMN).

Fig. 6C

Ventral genes (genes induced by Shh at 12h or 24h) and dorsal genes (genes repressed by Shh at 24h) are defined in paragraph Fig. 6B.

Fig. S3B,C – Table S5, sheet FigS3

To identify early and late FP genes, gene lists S3-1 (higher in 36h-FP than 36FP-p3) and S3-2 (higher in 60h-FP than 60FP-p3) were combined. To generate the heatmap, the average expression values from the replicate samples (S3-3) corresponding to 0h-FP, 9h-FP, 18h-FP, 36h-FP, 60h-FP conditions were normalized across all conditions (z-scores). FP genes were classified into “FP-early” (genes induced to maximum levels at 18 or 36h) and “FP-late” (genes induced to maximum levels at 60h) based on the kinetics of induction. For full list details, including classification, see Table S1, sheet S3B,C.

Fig. S4A - Table S5, sheet FigS4A.

To define genes induced by Shh at either 12h or 24h in ventral progenitors, two gene lists were combined: S4-1 (higher in 24h-p3 than 24h-dorsal(30RA)) and S4-2 (higher in 24h-pMN than 24h-dorsal(300RA)). To generate the heatmap, the average expression values from the replicate samples (S4-3) corresponding to 0h-FP, 12h-FP, 24h-FP, 36h-FP, 0h-p3, 12h-p3, 24h-p3, 36h-p3, 0h-pMN, 12h-pMN, 24h-pMN, 36h-pMN samples were normalized across all conditions (z-scores). Genes were classified according their time of induction in FP, p3 or pMN progenitors into “Shh-induced-12h” and “Shh-induced-24h”. For full list details, including classification, see Table S1, sheet S4.

Fig. S4B-C
Shh-induced genes (genes induced by Shh at 12h or 24h) and Shh-repressed genes (genes repressed by Shh at 24h) are defined in paragraph Fig. 6B.

Supplemental References

Berger, M.F., Badis, G., Gehrke, A.R., Talukder, S., Philippakis, A.A., Peña-Castillo, L., Alleyne, T.M., Mnaimneh, S., Botvinnik, O.B., Chan, E.T., et al. (2008). Variation in homeodomain DNA binding revealed by high-resolution analysis of sequence preferences. Cell *133*, 1266–1276.

Chen, C.Y., and Schwartz, R.J. (1995). Identification of novel DNA binding targets and regulatory domains of a murine tinman homeodomain factor, nkx-2.5. J. Biol. Chem. *270*, 15628–15633.

Huang, D.W., Sherman, B.T., and Lempicki, R.A. (2009). Systematic and integrative analysis of large gene lists using DAVID bioinformatics resources. Nature Protocols *4*, 44–57.

Jolma, A., Yan, J., Whitington, T., Toivonen, J., Nitta, K.R., Rastas, P., Morgunova, E., Enge, M., Taipale, M., Wei, G., et al. (2013). DNA-binding specificities of human transcription factors. Cell *152*, 327–339.

Li, H., Handsaker, B., Wysoker, A., Fennell, T., Ruan, J., Homer, N., Marth, G., Abecasis, G., Durbin, R. (2009). 1000 Genome Project Data Processing Subgroup. The Sequence Alignment/Map format and SAMtools. Bioinformatics *25*, 2078–2079.

Mazzoni, E.O., Mahony, S., Iacovino, M., Morrison, C.A., Mountoufaris, G., Closser, M., Whyte, W.A., Young, R.A., Kyba, M., Gifford, D.K., et al. (2011). Embryonic stem cell-based mapping of developmental transcriptional programs. Nat. Methods *8*, 1056–1058.

Pepinsky, R.B., Zeng, C., Wen, D., Rayhorn, P., Baker, D.P., Williams, K.P., Bixler, S.A., Ambrose, C.M., Garber, E.A., Miatkowski, K., et al. (1998). Identification of a palmitic acid-modified form of human Sonic hedgehog. J. Biol. Chem. *273*, 14037–14045.

Poirier, K., Van Esch, H., Friocourt, G., Saillour, Y., Bahi, N., Backer, S., Souil, E., Castelnau-Ptakhine, L., Beldjord, C., Francis, F., et al. (2004). Neuroanatomical distribution of ARX in brain and its localisation in GABAergic neurons. Brain Res. Mol. Brain Res. *122*, 35–46.

Quinlan, A.R., Hall, I.M. (2010). BEDTools: a flexible suite of utilities for comparing genomic features. Bioinformatics *26*, 841–842.

Saeed, A.I., Sharov, V., White. J., Li, J., Liang, W., Bhagabati, N., Braisted, J., Klapa, M., Currier, T., Thiagarajan, M., et al. (2003). TM4: a free, open-source system for microarray data management and analysis. BioTechniques *34*: 374–378.

Williams, K.P., Rayhorn, P., Chi-Rosso, G., Garber, E.A., Strauch, K.L., Horan, G.S., Reilly, J.O., Baker, D.P., Taylor, F.R., Koteliansky, V., et al. (1999). Functional antagonists of sonic hedgehog reveal the importance of the N terminus for activity. J. Cell. Sci. *112 (Pt 23)*, 4405–4414.
