# Supplementary material for: Neural Progenitors Adopt Specific Identities by Directly Repressing All Alternative Progenitor Transcriptional Programs
Source: Dev Cell. 2016 Mar 21;36(6):639–53. doi: 10.1016/j.devcel.2016.02.013 (PMC4819439; doi:10.1016/j.devcel.2016.02.013)
Supplement: Document S2. Article plus Supplemental Information [file mmc7.pdf]

Developmental Cell

Article

Neural Progenitors Adopt Specific Identities by Directly Repressing All Alternative Progenitor Transcriptional Programs

Graphical Abstract

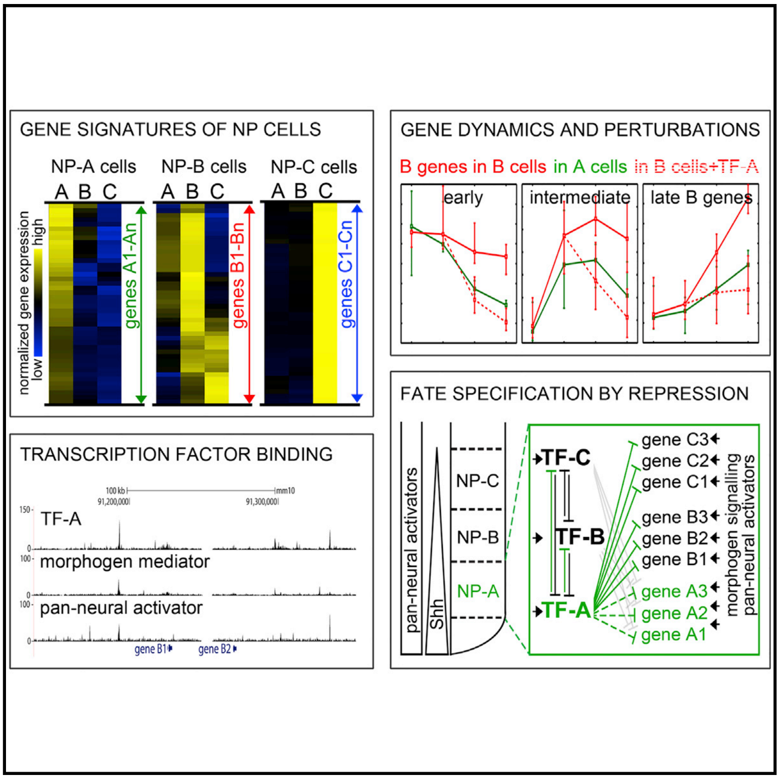

Highlights

- Specific vertebrate neural progenitor populations generated in vitro
- Gene expression dynamics, transcription factor binding assessed in neural progenitors
- Progenitor fate selected by repressors blocking entire programs of other identities
- Repressors counteract non-selective morphogen and pan-neural activatory inputs

Authors

Eva Kutejova, Noriaki Sasai, Ankita Shah, Mina Gouti, James Briscoe

Correspondence

james.briscoe@crick.ac.uk

In Brief

Neuronal subtype pattern in the vertebrate neural tube is determined by neural progenitor transcription factors (NP-TFs) expressed in stereotypic domains. Kutejova et al. show that achieving the correct pattern requires active repression of all but one fate by NP-TFs to counteract wide-ranging activating inputs from morphogen signaling and broadly expressed TFs.

Kutejova et al., 2016, Developmental Cell *36*, 639–653
March 21, 2016 ©2016 The Authors
http://dx.doi.org/10.1016/j.devcel.2016.02.013

Developmental Cell
Article

Neural Progenitors Adopt Specific Identities by Directly Repressing All Alternative Progenitor Transcriptional Programs

Eva Kutejova,[1] Noriaki Sasai,[1,2] Ankita Shah,[1,3] Mina Gouti,[1] and James Briscoe[1,*]
[1]The Francis Crick Institute, Mill Hill Laboratory, The Ridgeway, Mill Hill, London NW7 1AA, UK
[2]Present address: Developmental Biomedical Science, Graduate School of Biological Sciences, Nara Institute of Science and Technology (NAIST), 8916-5, Takayama-cho, Ikoma 630-0192, Japan
[3]Present address: Department of Animal and Avian Sciences, University of Maryland, College Park, MD 20742, USA
*Correspondence: james.briscoe@crick.ac.uk
http://dx.doi.org/10.1016/j.devcel.2016.02.013
This is an open access article under the CC BY license (http://creativecommons.org/licenses/by/4.0/).

SUMMARY

In the vertebrate neural tube, a morphogen-induced transcriptional network produces multiple molecularly distinct progenitor domains, each generating different neuronal subtypes. Using an in vitro differentiation system, we defined gene expression signatures of distinct progenitor populations and identified direct gene-regulatory inputs corresponding to locations of specific transcription factor binding. Combined with targeted perturbations of the network, this revealed a mechanism in which a progenitor identity is installed by active repression of the entire transcriptional programs of other neural progenitor fates. In the ventral neural tube, sonic hedgehog (Shh) signaling, together with broadly expressed transcriptional activators, concurrently activates the gene expression programs of several domains. The specific outcome is selected by repressive input provided by Shh-induced transcription factors that act as the key nodes in the network, enabling progenitors to adopt a single definitive identity from several initially permitted options. Together, the data suggest design principles relevant to many developing tissues.

INTRODUCTION

Pattern formation in developing tissues relies on the cells adopting one of several alternative fates. These decisions are determined by extrinsic signals, often in the form of morphogen gradients, and the transcriptional network that responds to the gradients. Together these form gene-regulatory networks (GRNs) that control gene expression and specify cell identity (Davidson, 2010).

In the vertebrate neural tube, the pattern of neuronal subtype generation is determined by the combinatorial activity of a set of transcription factors (TFs) expressed in neural progenitors (we term these transcription factors NP-TFs, and the network NP-GRN) (Alaynick et al., 2011; Dessaud et al., 2008; Jessell, 2000) (Figure 1A). The expression of NP-TFs, organized into stereotypic domains along the dorsal-ventral axis, is established progressively in response to anti-parallel morphogen gradients. In the ventral half of the neural tube, sonic hedgehog (Shh) signaling is associated with activation of ventral NP-TFs and simultaneous repression of NP-TFs characteristic of dorsal domains (Briscoe et al., 2000; Dessaud et al., 2008; Oosterveen et al., 2012, 2013; Peterson et al., 2012; Vokes et al., 2007). Many NP-TFs are able to act as Groucho/TLE-dependent repressors (Muhr et al., 2001) and pairs of NP-TFs expressed in adjacent domains cross-repress each other to form bistable switches that select the appropriate cellular identity (Balaskas et al., 2012; Briscoe et al., 2000; Novitch et al., 2001; Vallstedt et al., 2001). For example, Nkx2.2, expressed in p3 progenitors and required for V3 interneuron and visceral motor neurons (MNs) specification (Briscoe et al., 1999), is located ventrally to pMN progenitors, which express Pax6 and Olig2 (Alaynick et al., 2011) and generate somatic MNs (Novitch et al., 2001). Initially, Pax6 inhibits Nkx2.2 induction, allowing rapid induction of Olig2 by Shh signaling in presumptive p3 and pMN progenitor cells. Later, the induction of Nkx2.2, by continued Shh signaling, inhibits the expression of Pax6 and Olig2 in the p3 cells thereby delineating the p3/pMN boundary (Balaskas et al., 2012; Jeong and McMahon, 2005). The consequence is that p3 progenitors are located ventral to pMN progenitors. Similarly, Olig2 and Irx3, as well as Nkx6.1 and Dbx2, form bistable switches that demarcate additional boundaries in the ventral neural tube that are associated with the dorsal limits of MN and V2 neuron generation, respectively (Novitch et al., 2001; Sander et al., 2000; Vallstedt et al., 2001). In this way, the combination of the cross-repression and the response of NP-TFs to Shh signaling provide a mechanism to establish and position the discrete boundaries of gene expression domains (Balaskas et al., 2012; Briscoe et al., 2000).

In addition to repressing adjacent progenitor identities, however, forced expression of individual NP-TFs imposes the corresponding identity on progenitors throughout the neural tube (Briscoe et al., 2000; Muhr et al., 2001). This suggests a model in which NP-TFs repress the gene expression programs characteristic not only of adjacent but also of non-adjacent progenitor domains, in order to install the new transcriptional identity (Lee and Pfaff, 2001; Lee et al., 2004; Muhr et al., 2001). Whether

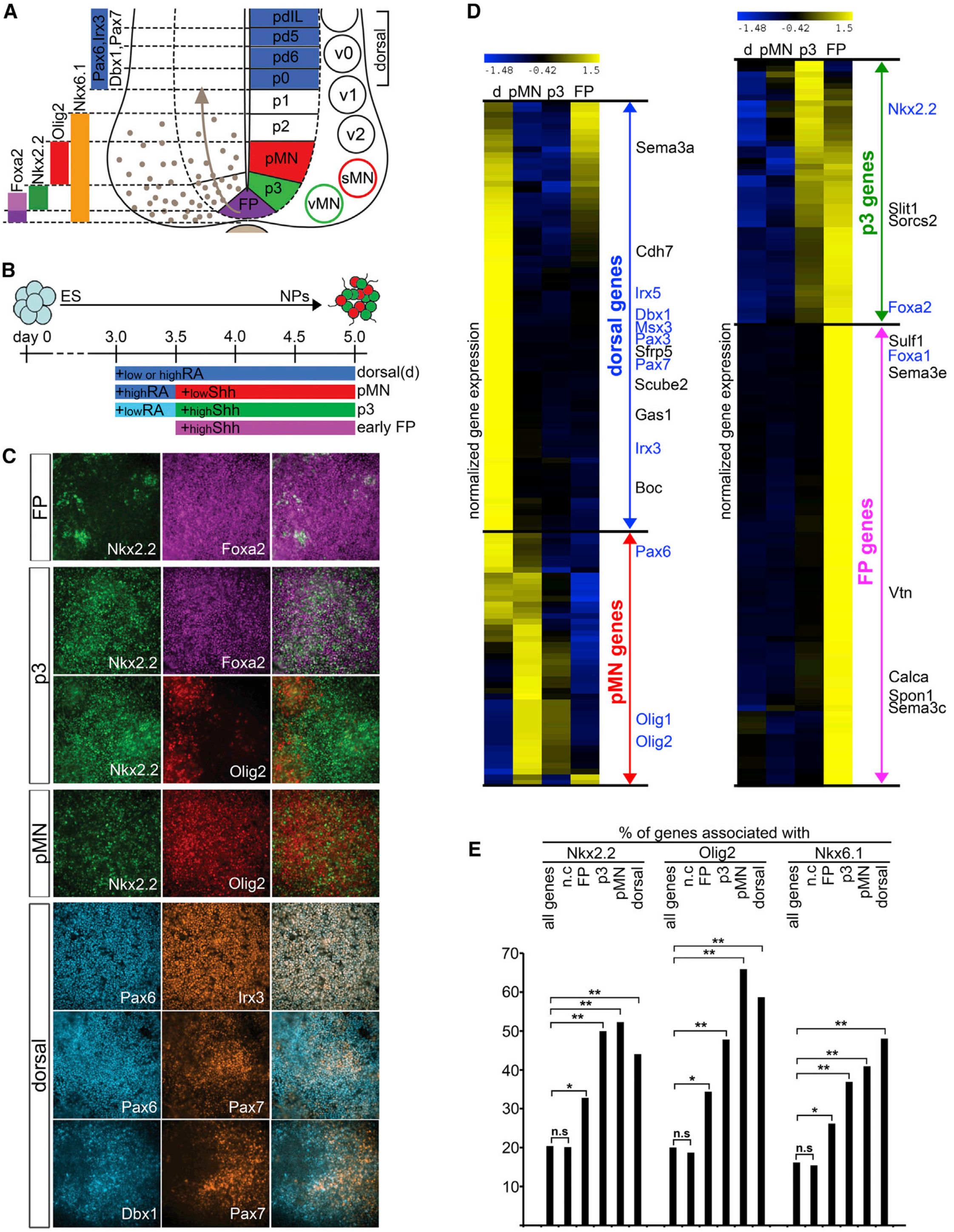

Figure 1. Nkx2.2, Olig2, and Nkx6.1 Bind to Loci Associated with Many of the Genes Differentially Expressed in Neural Progenitor Cells

(A) Schematic of the ventral neural tube. FP, p3, pMN, and dorsal progenitor domains are defined by the expression of Foxa2 (FP), Foxa2 and Nkx2.2 (p3), Olig2 (pMN), Pax6, Irx3, Pax7, Dbx1 (dorsal).

(B) Schematic of ESCs differentiated in monolayer culture into neural progenitors (iNPCs). ESCs were differentiated in monolayer in minimal N2B27 media, and RA and Shh were added at specified concentrations at the indicated times to induce FP, p3, pMN, and dorsal progenitor identities.

(C) The expression of the indicated neural progenitor transcription factors (NP-TFs) in iNPCs at day 5. Merged images are shown on the right panel. The in vitro conditions recapitulate the in vivo expression profiles of NP-TFs in FP, p3, pMN, and a mixture of dorsal progenitor identities (p0 to pd5).

(legend continued on next page)

this is achieved by repressing domain-specific TFs or by direct regulation of all domain-specific genes is unclear. Our current knowledge of the NP-GRN is based principally on genetic perturbation experiments and inference from a small number of genes and associated *cis*-regulatory elements (CREs) (Dessaud et al., 2008; Oosterveen et al., 2012, 2013; Peterson et al., 2012; Vokes et al., 2007). The pattern of genomic deployment of NP-TFs, and their direct effects on target genes, is largely unknown.

To distinguish between direct and indirect repression of domain-specific transcriptional programs and to determine how repression of adjacent and non-adjacent identities contributes to progenitor type specification, we determined the gene expression programs of progenitor cells corresponding to specific domains. Focusing on the ventral domains, we determined the genomic distribution of three NP-TFs, Nkx2.2, Olig2, and Nkx6.1 that collectively define the identities of p3, pMN, and p2 progenitors (Figure 1A). We correlated the genomic occupancy with the transcriptomes of defined progenitor populations following targeted perturbations of specific NP-TFs. These data indicate that ventral NP-TFs specify progenitor identities by directly repressing both the transcriptional determinants and progenitor-specific effector genes of adjacent and non-adjacent domains. Examination of the genomic binding of the pan-neural TF Sox2 (Bergsland et al., 2011; Oosterveen et al., 2012, 2013; Peterson et al., 2012) and the effector of hedgehog signaling Gli1 (Peterson et al., 2012) supports their direct involvement in the activation of neural progenitor genes and suggests that a substantial part of the positive input into the transcription of the ventral genes is provided directly by Shh signaling. Thus, the active repression of all genes specific for alternative progenitor identities is required to counteract wider-ranging activating inputs provided by broadly expressed TFs and broadly active mediators of morphogen signaling.

RESULTS

In Vitro Generation of Specific Neural Progenitor Identities

Systematic analysis of the neural progenitor GRN has been limited by the difficulty of isolating sufficient numbers of progenitor cells with defined identities. To circumvent this, we developed an in vitro system for inducing specific populations of neural progenitor cells (iNPCs) from undifferentiated embryonic stem cells (ESCs) (Figures 1B and 1C) (Andersson et al., 2006; Ying et al., 2003). By varying concentrations of two morphogens implicated in the specification of ventral neuronal subtypes, Shh and retinoic acid (RA) (Briscoe et al., 2001; Ericson et al., 1997; Novitch et al., 2003), we defined conditions that reproducibly generated progenitor populations with gene expression profiles characteristic of the floor plate (iNPC-FP) expressing Foxa2, the visceral MN-generating p3 domain (iNPC-p3) expressing Nkx2.2, somatic MN progenitors (iNPC-pMN) expressing Olig2 and dorsal neural progenitor populations (iNPC-D); the latter represent a mixture of p0 and several other dorsal cell types. All dorsal progenitors express Pax6 and Irx3 and subsets express Pax7 and/or Dbx1 (Figures 1A–1C). Comparison of the transcriptomes of dorsal, p3, pMN, and FP cells identified gene sets specifically expressed in each subset of progenitors (Figure 1D). These correlated well with known in vivo expression patterns (Table S1, sheet 1D) (Alaynick et al., 2011). Neural progenitor genes specific for different domains were enriched in TFs (18%) as well as glycoproteins, membrane-associated and secreted molecules related to the effector functions of the progenitor cells (54%) (Figure S1A and Table S1, sheet 1D).

NP-TF Binding Is Associated with Domain-Specific Genes

Discrete boundaries between progenitor domains are established by bistable switches formed by repressing pairs of NP-TFs expressed in adjacent domains (Balaskas et al., 2012; Briscoe et al., 2000; Novitch et al., 2001; Vallstedt et al., 2001). Whether the choice of corresponding cell fates involves only reciprocal repressive interactions between domain-specific NP-TFs or also entails the direct inhibition of all genes specific for the other domains is unclear. To identify the direct regulatory activities of the NP-TFs that define specific progenitor domains, we used chromatin immunoprecipitation sequencing (ChIP-seq) to examine the genome-wide binding profiles of Nkx2.2 (expressed in p3 progenitors), Olig2 (expressed in pMN), and Nkx6.1 (expressed in both p3 and pMN, ChIP performed from p3). We identified 2,000–3,000 binding sites for each TF corresponding to the highest signals, and de novo motif discovery (Gupta et al., 2007; Heinz et al., 2010) within these regions recovered consensus motifs consistent with the known sequence preference of each TF (Figure S1B). NP-TFs bound predominantly to distal CREs, rather than directly at gene promoters (Figures S1C and 3I). By associating each occupied site with its neighboring genes, we found a marked enrichment of NP-TF binding not only around NP-TFs but also associated with a large fraction of other genes that exhibited differential regulation in iNPCs (Figure 1E). This suggested a network of transcriptional interactions more complex than required if the cross-repressive interactions were limited to NP-TFs expressed in adjacent domains. The broad deployment of NP-TFs next to the genes with which they showed mutual exclusion provided evidence that progenitor fate specification involved the direct repression of the entire transcriptional program of other domains. To test this hypothesis we first investigated the regulation of the Nkx2.2 targets.

Nkx2.2 Binds and Represses Transcriptional Programs of Progenitors Adjacent to the p3 Domain

Nkx2.2 specifies the program of p3 progenitors (Briscoe et al., 1999). This domain is flanked dorsally by pMN, expressing the pMN-defining NP-TF Olig2 (Alaynick et al., 2011; Novitch et al.,

(D) Transcriptome analysis of iNPCs defines gene expression signatures for each progenitor identity. Heatmaps of gene expression levels in the indicated progenitor types highlights the unique signature of the distinct progenitor subtypes. Transcription factors specifying the different domains are indicated in blue, transmembrane or secreted molecules with known expression pattern are marked in black. See also Table S1, sheet 1D.

(E) Nkx2.2, Olig2, and Nkx6.1 peaks are enriched next to genes differentially expressed in iNPCs. The barchart compares the percentage of genes associated with binding of the indicated NP-TFs for genes upregulated in specific iNPC populations with all genes or genes that do not change in expression in iNPCs (n.c). $^{**}p(\chi^2) < 0.001$, $^{*}p(\chi^2) < 0.025$, n.s, non-significant. See also Figure S1.

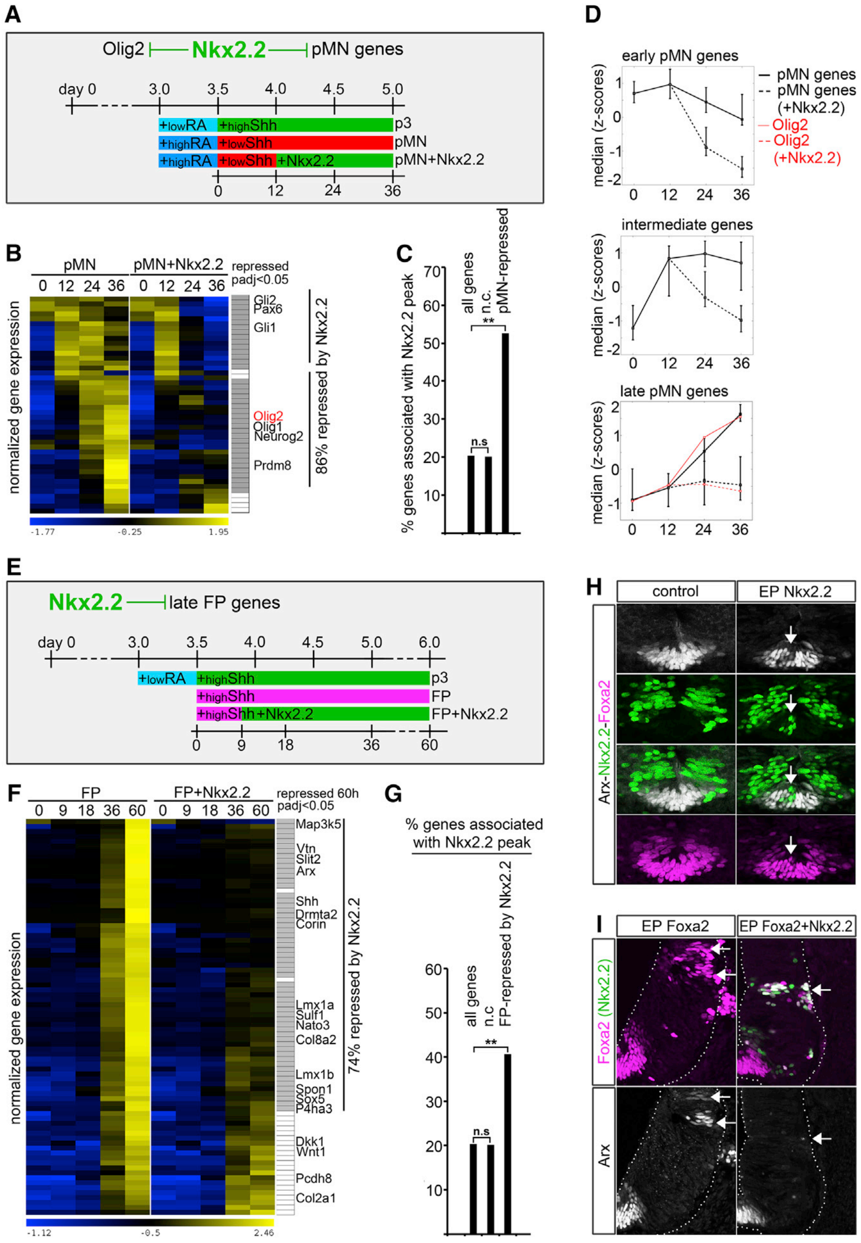

Figure 2. Nkx2.2 Directly Represses Genes Expressed in Adjacent Domains

(A–D) Nkx2.2 directly represses pMN genes independently of Olig2 downregulation. (A) Schematic of the experimental rationale. To test whether Nkx2.2 represses both Olig2 and other pMN genes directly, ESCs were differentiated to p3 or pMN identity using the indicated schedules of RA and Shh treatment. Ectopic Nkx2.2 expression was induced in pMN progenitors by the addition of doxycycline 12 hr after Shh was added (pMN + Nkx2.2). (B) Heatmap comparing the time course of gene expression in pMN and pMN + Nkx2.2 iNPCs. Genes characteristic of pMN identity defined in Figure 1D as "pMN" (red) were expressed higher in pMN compared with p3. The induction of Nkx2.2 in pMN conditions repressed 86% of these genes. Filled gray boxes adjacent to the heatmap indicate genes significantly downregulated compared with pMN (padj < 0.05). Genes with known pMN-restricted (pMN > p3) expression are indicated. See also Table S1, sheet 2B. (C) Nkx2.2 binding is associated with many pMN genes that are repressed by Nkx2.2 induction. 53% of the pMN genes repressed by Nkx2.2 induction are associated with the binding of Nkx2.2. By contrast only 20% of all genes or genes that do not change in expression in neural progenitors have associated binding sites (n.c). **p(X^2) < 0.001, n.s, non-significant. (D) Median expression levels of genes induced at early (n(genes) = 5), intermediate (n(genes) = 16) and late (n(genes) = 17) times in pMN cells (solid lines). The lines represent the median values of the indicated groups of genes and the error bars correspond to the 10th and 90th percentile values of each group. Most genes are induced prior to Olig2 (red line), which is induced relatively late. Both the early and intermediate classes of genes are downregulated (dotted lines) by the time Olig2 is fully induced (24 hr).

(E–I) Nkx2.2 directly represses late FP genes. (E) Schematic of experimental rationale. To test whether Nkx2.2 inhibits the elaboration of FP identity by repressing genes induced at late times in FP differentiation, ESCs were induced to p3 or FP identity. Ectopic Nkx2.2 expression was induced in FP progenitors by doxycycline addition 9 hr after Shh treatment. (F) Heatmap comparing the time course of gene expression in FP and FP + Nkx2.2 iNPCs. Genes characteristic of definitive FP identity (late FP genes) were selected by identifying genes expressed at higher levels in FP than in p3 progenitors, induced to maximum levels at 60 hr (Figure S3). Genes with known FP-restricted expression are indicated. The induction of Nkx2.2 in FP conditions repressed 74% of these genes. Filled gray boxes adjacent to the heatmap indicate genes significantly downregulated in FP + Nkx2.2 compared with FP (padj < 0.05) at 60 hr. See also Figure S3 and Table S1, sheet 2F. (G) Nkx2.2 binding is associated with many of the FP genes that are repressed by Nkx2.2 induction. 40% of the FP genes repressed by Nkx2.2 induction are associated with the binding of Nkx2.2. **p(χ^2) < 0.001, n.s, non-significant. (H) Nkx2.2 represses the late FP marker Arx in vivo. Nkx2.2 (green) was electroporated ventrally in ovo into the chick neural tube at HH8 and the expression of Arx (white) and Foxa2 (purple) was analyzed 48 hr later. At the ventral midline of experimental sample, Nkx2.2-expressing cells contained substantially lower levels of Arx expression (arrows). (I) Nkx2.2 acts downstream or in parallel to Foxa2 to repress late FP marker expression in vivo. Foxa2 or Foxa2 and Nkx2.2 were electroporated laterally into the chick neural tube at HH12 and the expression of Arx was analyzed 48 hr later. Foxa2 (purple) induces Arx expression (white) in intermediate/dorsal progenitors, in a cell-autonomous manner (arrows). By contrast, co-expression of Nkx2.2 (green) abolishes Foxa2-induced induction of Arx (arrow). The dotted lines indicate the outlines of the neural tube.

2001); and ventrally by FP, which expresses the NP-TF Foxa2, and later Nato3 and Arx (Mansour et al., 2014; Ribes et al., 2010; Sasai et al., 2014). We asked whether Nkx2.2 alone was sufficient to downregulate the expression of pMN-specific genes and whether its effects on gene expression were mediated by direct repression of all pMN genes or indirectly by regulating Olig2 expression. For this purpose, we developed an ESC line containing an inducible Nkx2.2 cDNA under the control of tetracycline-regulatory elements (ESC-iNkx2.2; Figure S2) (Gouti and Gavalas, 2008; Selfridge et al., 1992). Induction of Nkx2.2 expression under iNPC-pMN conditions led to the rapid downregulation of 86% of pMN-specific genes, including Olig2 (Figures

2B and S2, and Table S1, sheet 2B). Analysis of the genomic occupancy of Nkx2.2 in p3 cells revealed that Nkx2.2 could be detected at more than 50% of these genes (Figure 2C), suggesting that Nkx2.2 acts as a direct repressor of many pMN-specific genes. Consistent with a direct repressive role of Nkx2.2, a large fraction of pMN-specific genes were induced prior to onset of Olig2 expression (ruling out an involvement of Olig2 in the activation of these genes) and repressed before Olig2 was fully upregulated in pMN cells (Figure 2D).

Nkx2.2 is transiently expressed in cells that become FP and is involved in FP specification (Jeong and McMahon, 2005; Lek et al., 2010). However, Nkx2.2 is downregulated in FP as development proceeds (Ribes et al., 2010). Analysis of gene expression in iNPC-FP cells identified an expression signature characteristic of definitive FP, including the expression of Nato3 (Mansour et al., 2014) and Arx (Ribes et al., 2010) (Table S1, sheet 2F). This signature emerged between 36 and 60 hr, as Nkx2.2 was downregulated (Figures S3A–S3C). Artificially sustaining expression of Nkx2.2 in iNPC-FP progenitors derived from ESC-iNkx2.2 cells abrogated the upregulation of 74% of the definitive FP genes (Figure 2F). A large fraction of the genes inhibited by Nkx2.2 were associated with Nkx2.2-binding events (Figure 2G), consistent with a direct repressive activity. Thus, following the requirement for Nkx2.2 activity in the initiation of FP differentiation, through the repression of Pax6, Gli3, and Olig2 (Lek et al., 2010), Nkx2.2 must be downregulated to allow the full elaboration of FP identity.

We confirmed the repressive effect of Nkx2.2 on induction of genes characteristic of definitive FP using in ovo electroporation of chick embryos. Forced continuous expression of Nkx2.2 cell autonomously prevented upregulation of the FP marker Arx (Figure 2H). Moreover, co-electroporation of Nkx2.2 blocked Foxa2-dependent induction of Arx (Figure 2I), ruling out an indirect mode-of-action of Nkx2.2 through the regulation of the FP determinant Foxa2. Taken together, these data provide evidence that, in addition to repressing the NP-TFs of adjacent domains, Nkx2.2 directly inhibits a major fraction of the entire transcriptional programs of both pMN and FP, by binding at CREs linked to the genes expressed in these domains.

Nkx2.2 and Nkx6.1 Bind and Repress Genes Specific to the Programs of Distant Progenitor Domains

As well as genes associated with the adjacent pMN and FP progenitor domains, we noticed Nkx2.2 occupancy was enriched around genes expressed in progenitors of non-adjacent dorsal domains (Figure 1E). These included genes characteristic of several distinct progenitor domains present within the iNPC-D population (Figure 3D and Table S1, sheets 1D and 3B), suggesting that Nkx2.2 may also play a direct role in the regulation of gene expression programs of multiple non-adjacent domains. To test whether Nkx2.2 could directly repress these genes, we used ESC-iNkx2.2 cells to induce Nkx2.2 expression in iNPC-D cells. In these cells, induction of Nkx2.2 strongly repressed 53% of the genes specifically expressed in dorsal progenitors (Figure 3B). The remainder of the dorsal genes were affected to a lesser extent.

Since Nkx6.1 is expressed with Nkx2.2 in p3 cells, as well as in pMN and FP cells (Briscoe et al., 2000; Sander et al., 2000), we hypothesized that it might cooperate with Nkx2.2 in the repression of dorsal gene expression programs. We constructed additional cell lines that allow the inducible expression of either Nkx6.1 (ESC-iNkx6.1) or Nkx2.2 and Nkx6.1 together (ESC-iNkx2.2-2A-Nkx6.1 and ESC-iNkx6.1-2A-Nkx2.2) (Figure S2). Induction of Nkx6.1 alone in iNPC-D cells led to the downregulation of a further $\sim$12% of dorsally expressed progenitor genes (Figure 3B). By contrast, co-induction of Nkx2.2 and Nkx6.1 led not only to a more pronounced inhibition of genes that were repressed by Nkx2.2 alone, but also to the downregulation of an additional set of NPC-D genes (Figure 3B). This indicates that these NP-TFs act in a complementary manner. In total, almost 90% of the combined gene expression programs of dorsal domain progenitors were repressed (Figure 3B). The binding of Nkx2.2 and Nkx6.1 was significantly enriched around genes that were inhibited by expression of Nkx2.2 and/or Nkx6.1 (Figures 3C and 3I), in line with a direct repressive activity at these genes. This implies that domain-specific gene expression programs are installed by a repressive mechanism directly acting on all genes specific for alternative fates.

A corollary of this is that to protect domain-specific gene expression, an NP-TF must repress not only the NP-TFs expressed in adjacent progenitor domains, but NP-TFs of all other domains. Systematic analysis of the effects of forced Nkx2.2 and Nkx6.1 expression confirmed that this is the case: in addition to Pax6 and Olig2, Nkx2.2 together with Nkx6.1 repressed Irx3/5, Dbx1, Pax3/7, and Msx3 NP-TFs expressed in dorsal progenitor domains (Figure 3D) (Alaynick et al., 2011). Moreover, the repression of all transcriptional determinants prevents the indirect upregulation of NP-TFs from non-adjacent domains that would otherwise result from the serial repression of only the NP-TFs in adjacent domains. Consistent with this, ectopic expression of a subset of non-adjacent dorsally expressed NP-TFs has been documented in the p3 domain of embryos lacking Nkx6.1 (Sander et al., 2000).

Olig2 Substitutes for Nkx2.2 in MN Progenitors

The broad repression of inappropriate-domain transcriptional programs by NP-TFs implies that each domain-specific gene must be repressed by NP-TFs from multiple domains. The repression by Nkx2.2 of genes normally restricted to dorsal neural progenitors raises the question of how these genes are repressed in the pMN domain (where Nkx2.2 is not expressed). We asked whether the pMN NP-TF Olig2 substituted for Nkx2.2. We constructed a cell line that allows the inducible expression of Olig2 (ESC-iOlig2) (Figure S2). Induction of Olig2 in iNPC-D cells downregulated expression of 56% of dorsal genes, including genes specific for non-adjacent dorsal domains that are repressed by Nkx2.2 in p3 progenitors (Figure 3G). Consistent with a direct repressive role, analysis of the genomic locations of Olig2 binding indicated that it is associated with a large proportion of dorsal-specific genes (Figure 3H). Despite their different DNA-binding specificities (Figure S1B), in many cases Nkx2.2 and Olig2 appear to occupy the same CREs to repress the dorsal genes (Table S4 and Figure 3I). Thus, to repress domain-inappropriate gene expression programs Nkx2.2 and Olig2 appear to act in part through shared CREs. This contrasts with Nkx6.1 and Nkx2.2, for which only 11% of CREs associated with dorsal genes repressed in p3 that are bound by Nkx2.2 are also occupied by Nkx6.1 (Table S4 and Figure 3I).

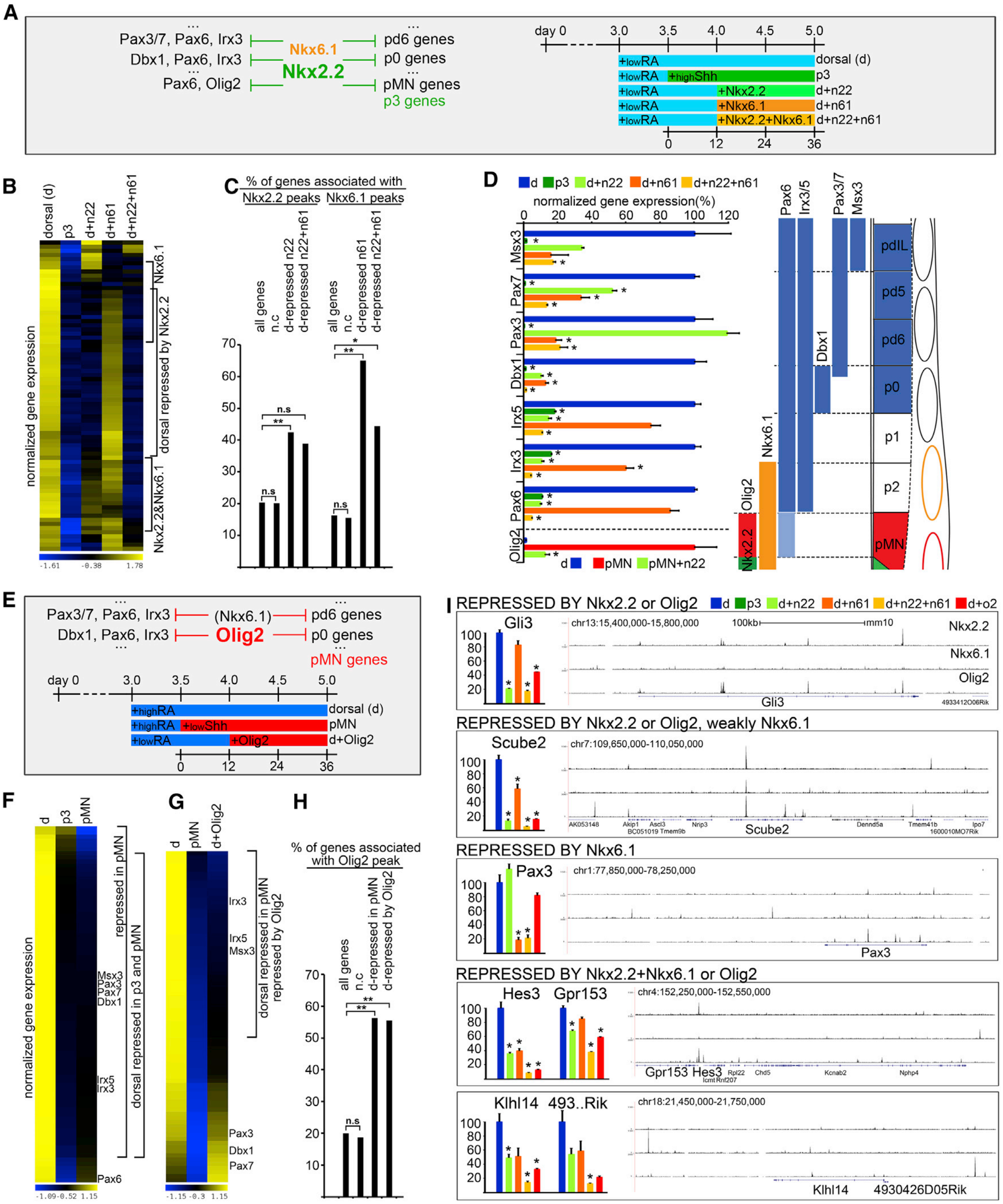

Figure 3. Nkx2.2, Nkx6.1, and Olig2 Repress Genes of Non-adjacent Progenitors

(A–D) Nkx2.2 together with Nkx6.1 repress non-adjacent dorsal identities. (A) Schematic of the experimental rationale. To test the activity of Nkx2.2 and Nkx6.1 in non-adjacent progenitor domains, ectopic Nkx2.2 (n22), Nkx6.1 (n61), or Nkx2.2 and Nkx6.1 (n22 + n61) were induced in iNPC-D cells and their transcriptomes compared with iNPC-D and iNPC-p3 cells. (B) Analysis of the transcriptomes of iNPC-D cells after ectopic induction of Nkx2.2 and/or Nkx6.1 indicated the repression of subsets of genes normally characteristic of dorsal progenitors. Genes characteristic of dorsal progenitors are defined as "dorsal" (blue) in Figure 1D. These were selected using the criteria of higher expression levels in dorsal progenitors compared with p3 and pMN cells at 36 hr. The induction of Nkx2.2 alone was sufficient to repress many of these genes; induction of Nkx6.1 repressed a smaller, partially overlapping subset of genes; the combination of Nkx2.2 and Nkx6.1 repressed the majority of iNPC-D-specific genes. See also Table S1, sheet 3B. (C) ChIP-seq data indicate that binding of Nkx2.2 and Nkx6.1 is enriched next to many of the iNPC-D genes repressed by Nkx2.2 and/or Nkx6.1 compared with genes that are not differentially expressed in neural progenitors. **p(χ^2) < 0.001. *p(χ^2) < 0.01. n.s, non-significant. (D) Analysis of a subset of NP-TFs specific for distinct dorsal domains (see adjacent diagram). Nkx2.2 and/or Nkx6.1 repress these NP-TFs. In some cases both Nkx2.2 and Nkx6.1 repress expression in iNPC-D cells. However, Nkx6.1 but not Nkx2.2 repress Pax3, whereas Nkx2.2 but not Nkx6.1 repress Irx3, Irx5, and Pax6. *padj < 0.05.

(legend continued on next page)

Repressor Activity of NP-TFs Is Sufficient for Patterning Activity

Nkx2.2 and some of the other NP-TFs can negatively regulate transcription through their interaction with Groucho/TLE-dependent transcriptional co-repressors (Muhr et al., 2001). To test whether repressor activity is sufficient to specify fully progenitor identity, we constructed an ESC line that inducibly expressed a chimeric protein consisting of the Nkx2.2 DNA-binding homeodomain fused to a well-characterized but unrelated Groucho/TLE-binding repressor domain (Muhr et al., 2001) (derived from the Drosophila Engrailed protein) — ESC-iNkx2.2HD-EnR (Figure S2). Expression of this protein in either iNPC-D or iNPC-pMN cells was sufficient to downregulate domain-specific programs and activate the p3 program (Figures 4B and 4C), mimicking the effect of the full-length protein. Thus, at least in the case of Nkx2.2, repressor function is the major, and probably only, activity necessary for establishing domain-specific gene expression program.

Strikingly, however, we detected binding of Nkx2.2 associated with genes that are expressed in the p3 domain (Figure 1E). If Nkx2.2 acts as a repressor, why are these genes not repressed? Due to the progressive and asynchronous differentiation of neural progenitors (Kicheva et al., 2014) this binding could correspond to the highly dynamic genes marking the small population of cells in transition from progenitor to postmitotic state. Thus, a fraction of the genes identified as "p3" may be actively repressed in progenitors but de-repressed on neuronal differentiation. Alternatively, it is possible that Nkx2.2 functions as a repressor of genes that are expressed in p3 progenitors, but this repressive activity is overpowered by positive inputs. To investigate this, we focused on a subset of genes that are repressed in pMN but expressed at some level in p3 but at higher levels in FP, which at 36 hr has reduced levels of Nkx2.2 compared with p3 (Table S1, sheet 1D, Figure S3A). As expected, ectopic induction of Nkx2.2 in pMN markedly increased the level of expression of these genes, consistent with the ability of Nkx2.2 to promote a p3 identity (Figure 5B). By contrast, however, in FP cells instead of further boosting their expression, as would be expected if Nkx2.2 functioned as an activator of these genes, induction of Nkx2.2 attenuated the expression of almost half of these genes (44%) (Figures 5B), and 72% of the downregulated were associated with Nkx2.2 binding (Figure 5C). This suggests Nkx2.2 represses a subset of p3 genes, albeit incompletely, and that this repression is overcome by activatory input in p3 cells. Consistent with weaker, but detectable, repressive effect of Nkx2.2 on p3 genes, forced expression of Nkx2.2 repressed genes characteristic of FP/p3 identity to a lesser extent than genes specific of dorsal or pMN progenitors (Figure 5D).

Analysis of Olig2 genomic occupancy also revealed binding associated with genes expressed in the pMN domain (Figure 1E). Even though Olig2 represses dorsal and p3 genes to promote pMN gene expression (Balaskas et al., 2012; Mizuguchi et al., 2001; Novitch et al., 2001; Zhou et al., 2001), inducing Olig2 expression in iNPCs exposed to Shh did not increase the expression of pMN-specific genes, as might be expected (Figure 5F). On the contrary, it further reduced the expression of a large fraction (63%) of genes normally expressed in pMN progenitors, implying that Olig2 negatively regulates their expression. These included both pMN-specific genes (Figure 5F) and genes expressed in multiple ventral domains (Figure 5G), such as Nkx6.1 and Nkx6.2. Eighty percent of the downregulated genes were bound by Olig2 (Figure 5H). Taken together these data suggest that the sole presence of a repressor is not sufficient to predict whether a gene will be repressed or not. Instead, the combination of positive and negative inputs must determine the response of a gene. In this way a gene is expressed in domains where the activatory inputs dominate the repressive ones. Moreover, the data further imply that the levels of some NP-TFs (e.g., Olig2) must be kept under tight control to allow for the expression of domain-specific genes. Accordingly, Olig2 transcription has been reported to oscillate (Imayoshi et al., 2013), providing a mechanism to yield low protein levels.

Repressive Activity of NP-TFs Is Integrated with Broadly Acting Positive Inputs

If NP-specific TFs act only as repressors, how are neural progenitor genes activated? We focused on the expression of genes specific for ventral domains, and asked whether morphogen signaling, in the form of Shh-induced Gli activity, might directly promote their expression. Using our gene expression data as a reference (Figure S4A), we reanalyzed the binding of Gli1 in neural progenitor cells (Peterson et al., 2012). Strikingly, direct Gli binding was detectable and highly enriched at a large fraction of genes that were upregulated in p3, pMN and early FP progenitors (Figure 6B). The directly bound genes comprised not only ventral NP-TFs, but 73% of the genes upregulated rapidly by Shh exposure and 46% of those that respond on a slower timescale. By contrast, only 11% of dorsal domain-specific genes, which are downregulated in response to Shh, were associated with direct Gli binding (Figure 6B). In addition to Gli proteins, SoxB proteins, which are expressed in all progenitor cells, have been suggested

(E–G) Olig2 represses dorsal neural progenitor genes in pMN cells. (E) Schematic of the experimental rationale. To test whether Olig2 contributed to the repression of dorsal genes in pMN progenitors, Olig2 was ectopically induced in iNPC-D cells and the transcriptomes of these cells compared with iNPC-pMN progenitors. (F) Heatmap comparing the transcriptomes of iNPC-D, iNPC-pMN, and iNPC-p3 cells. Most of the dorsal genes that are repressed in p3 cells are also repressed in pMN progenitors. See also Table S1, sheet 3F. (G) Comparison of the transcriptomes of iNPC-D and iNPC-pMN with iNPC-D in which Olig2 had been induced. Olig2 is sufficient to downregulate 56% of the dorsal genes normally repressed in pMN at 36 hr. See also Table S1, sheet 3G.

(H) ChIP-seq analysis confirms that Olig2 binds next to 56% of the dorsal genes downregulated in pMN and repressed by Olig2. $^{**}p(\chi^2) < 0.001$. n.s., non-significant.

(I) Examples of ChIP-seq tracks for Nkx2.2 (top), Nkx6.1 (middle), and Olig2 (bottom) for the Nkx2.2 and/or Nkx6.1 and/or Olig2 repressed genes. Nkx2.2 and Olig2 bind to multiple intronic CREs of Gli3. Nkx2.2, Olig2, and Nkx6.1 bind to intronic CRE of Scube2. Nkx6.1 binds two intergenic enhancers to repress Pax3. Two clusters of genes repressed cooperatively by Nkx2.2 and Nkx6.1 are bound by either Nkx2.2 (Hes3 and Gpr153) or Nkx6.1 (Klhl14 and 4930426D05Rik) in their vicinity, whereas the other NP-TFs bind further away, separated by several genes that are either not expressed or not differentially regulated in neural progenitors. Thus, genes repressed strongly by Nkx2.2 and weakly by Nkx6.1 seem to be regulated through partially shared CREs (Scube2), whereas genes that require both Nkx2.2 and Nkx6.1 for full repression are regulated through exclusively separate CREs (Table S4). Note also that Nkx2.2 and Olig2 often share regulatory elements controlling expression of dorsal genes. *padj < 0.05.

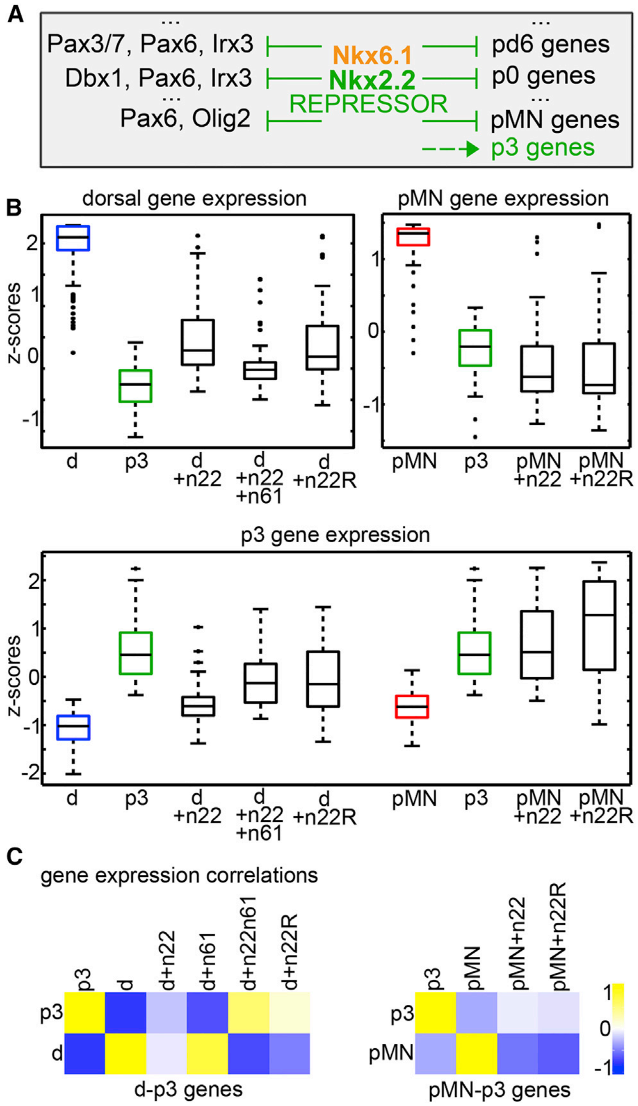

Figure 4. Regulation of Gene Expression Programs by NP-TFs Is Mediated by Repression

(A–C) Nkx2.2 represses dorsal and pMN genes to induce p3. (A) Experimental rationale: to test whether repression of dorsal and pMN genes by Nkx2.2 is sufficient to allow the de-repression of p3-specific genes, a dominant inhibitory version of Nkx2.2 (n22R) was induced in iNPC-D or iNPC-pMN cells and the resulting transcriptome changes analyzed. (B) Nkx2.2HD-EnR is sufficient to repress a large fraction of dorsal and pMN genes and to induce p3 identity. Three groups of genes defined in Figure 1D were analyzed: "dorsal" genes (blue) that were expressed higher in iNPC-D than iNPC-p3 or iNPC-pMN, "pMN" genes (red) that were expressed higher in iNPC-pMN than iNPC-p3, and "p3" genes (green) expressed at higher levels in iNPC-p3 than iNPC-D or iNPC-pMN, expressed in p3 only or in p3 and FP progenitors. The boxplots correspond to the normalized expression levels of these genes in each of the conditions. A large fraction of dorsal and pMN genes was repressed in d + n22R and pMN + n22R cells. Conversely, a large fraction of p3 genes were induced in these progenitors, similar to the progenitors generated by ectopic induction of Nkx2.2 (d + n22, pMN + n22) or Nkx2.2 and Nkx6.1 (d + n22n61). (C) Consistent with B, Nkx2.2 and Nkx2.2HD-EnR induced a p3 gene signature when overexpressed in dorsal or pMN cells. Cross-correlation of dorsal and p3 genes (Pearson's correlation coefficient) between iNPC-D, iNPC-p3, and iNPC-D cells in which Nkx2.2, Nkx6.1, Nkx2.2/Nkx6.1, or Nkx2.2HD-EnR had been induced indicate that Nkx2.2HD-EnR induces a transcriptome signature similar to p3 cells. Similar cross-correlations of gene expression in pMN and p3 progenitors indicate that the induction of Nkx2.2 and Nkx2.2HD-EnR induce a p3 signature in iNPC-pMN.

to act as positive regulators of neural expressed genes (Bailey et al., 2006; Bergsland et al., 2011; Oosterveen et al., 2012, 2013; Peterson et al., 2012). Consistent with this role, we also detected Sox2 binding next to both dorsally and ventrally expressed genes (Figure 6C). These data suggest that Shh signaling, together with broadly expressed transcriptional activators, directly activate the bulk of the genes that comprise the transcriptional programs of ventral progenitor domains. This includes the identity-defining NP-TFs that directly repress genes and domain-specific TFs belonging to the alternative fates. Consistent with this, early and intermediate pMN genes are simultaneously activated in both p3 and pMN conditions and then repressed in p3 by Nkx2.2 at 24 hr (Figure 6D).

To investigate how the positive input is integrated with the repressive activity of NP-TFs we focused on several previously tested CREs associated with ventral NP-TFs (Nkx2.2, Olig2, Nkx6.1, Nkx6.2) (Oosterveen et al., 2012, 2013; Peterson et al., 2012). Binding of Sox and of Gli proteins to these CREs have been demonstrated and implicated in their activation. The domain-restricted activity of these CREs has been attributed to NP-TFs. Consistent with this prediction, binding of one or more NP-TFs was observed on each of the CREs (Figures S5A–S5D) and the binding negatively correlated with the activity of the enhancer. Similar to other developmental systems (Barolo, 2012; Levine, 2010), both negative and positive inputs regulating the expression of a gene appeared distributed over multiple CREs and often redundant. Accordingly, analysis of the locations of Gli1, Nkx2.2, Olig2, Nkx6.1, and Sox2 binding suggested that most Shh-regulated genes (60%) were associated with three or more distinct CREs. Despite the large fraction of Shh-induced genes bound by Gli1 that were associated with Sox2 binding (63%), there was only limited co-occupancy of Gli and Sox at the same CRE (~20% of genes and CREs bound by Gli1, Figure S5A). Most (88%) of Shh-regulated genes that associate with Sox2/Gli1 or Sox2 binding were also associated with the binding of an NP-TF repressor. However, only 40% of the Sox2- or Gli1-bound CREs were also bound by one or more of the NP-TFs and only 22% of NP-TF-bound elements were associated with activator binding (Figures S4B and S4C). Consistent with redundant regulation, for half of genes associated with the binding of an NP-TF there were two or more CREs bound by the same protein. Taken together, therefore, these data suggest a mechanism in which the response of a gene is determined by multiple and probably partially redundant CREs that integrate the repressive activity of NP-TFs with the broad activity of morphogen mediators and transcriptional activators. This provides a means to interpret morphogen input and select a single and appropriate progenitor identity for the position in the neural tube.

DISCUSSION

The mechanistic strategies of the transcriptional network underlying neural progenitor differentiation have been proposed from genetic manipulations of specific genes and CREs (Oosterveen et al., 2012, 2013; Peterson et al., 2012). Here, by combining

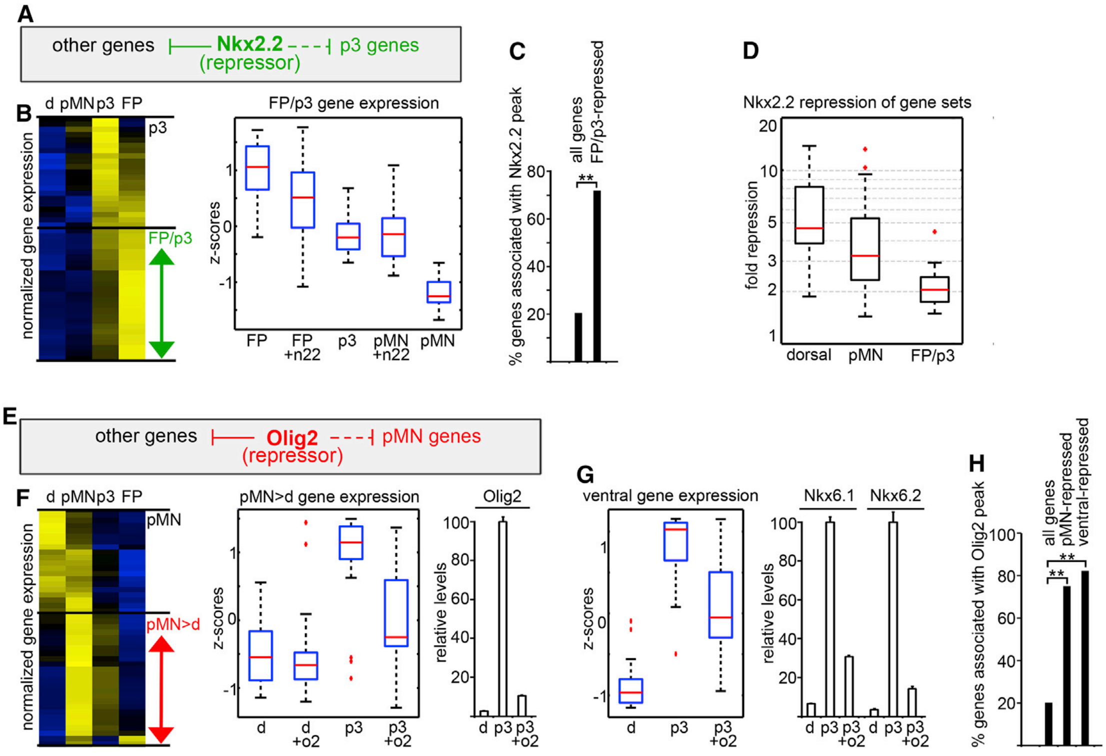

Figure 5. Gene Regulation Is Combinatorial

Genes are expressed where positive inputs overcome repressive inputs.

(A) Nkx2.2 directly attenuates expression of a subset of p3 genes. Nkx2.2 was induced in FP (9 hr) and pMN (12 hr) cells. The expression of genes expressed higher in FP > p3 ≫ pMN/dorsal (FP/p3, green arrow and Table S1, sheet 1D) was examined at 36 hr.

(B and C) Nkx2.2 represses 44% of genes expressed higher in FP than p3 when expressed in FP cells (B) and binds in their proximity (C, 73%, **p(χ^2) < 0.001). Compare FP with FP + n22 and p3. At the same time, Nkx2.2 indirectly promotes expression of these genes when expressed in pMN cells (B). Compare pMN with pMN + n22 and p3. This suggests that these genes are repressed more efficiently by Olig2/Pax6 NP-TFs in pMN than by Nkx2.2 in p3. Replacement of Olig2/Pax6 by Nkx2.2 in p3 results in their induction by allowing the activatory inputs to dominate the repression.

(D) Nkx2.2 represses FP/p3 genes less strongly than it represses dorsal and pMN genes. The fold repression of genes defined as Nkx2.2-repressed (Figures 3B, 2B, and 5B) after the induction of Nkx2.2 in 36-hr dorsal/dorsal + Nkx2.2 (“dorsal”), 36-hr pMN/pMN + Nkx2.2 (“pMN”), 36-hr FP/FP + Nkx2.2 (FP > p3 ≫ pMN/d genes, “FP/p3”). Note that FP/p3 genes are repressed to a lesser extent than the other classes.

(E) Olig2 levels must be low to allow expression of pMN genes. Ectopic expression of Olig2 results in rapid and direct downregulation of most genes induced in pMN progenitors, including Olig2 itself, Nkx6.1 and Nkx6.2 NP-TFs.

(F) High levels of Olig2 repress pMN genes. Olig2 expression was induced in p3 cells at 12 hr and the expression of pMN-specific genes (genes expressed higher in pMN compared with both p3 and dorsal, pMN > d, red arrow and Table S1, sheet 1D) were analyzed at 24 hr. Compare p3 with p3 + o2. 64% of the genes were downregulated by Olig2, the majority (75%) was associated with Olig2 binding (H, **p(χ^2) < 0.001).

(G) Olig2 expression was induced in p3 cells at 12 hr and the expression of genes induced by Shh in both p3 and pMN was analyzed at 24 hr. 62% of the ventral genes were downregulated by Olig2, the majority (82%) were associated with Olig2 binding (H, **p(χ^2) < 0.001).

genome-wide RNA-seq and ChIP-seq analyses with targeted perturbation experiments, we test these ideas and provide evidence for a “selection by exclusion” mechanism that specifies a particular progenitor subtype identity from multiple permitted choices. This confirms and extends previous proposals (Bailey et al., 2006; Bergsland et al., 2011; Lee and Pfaff, 2001; Lee et al., 2004; Muhr et al., 2001; Oosterveen et al., 2012, 2013; Peterson et al., 2012) and is consistent with recent results reported in a parallel study (Nishi et al., 2015). Together the data reveal four design features of the GRN. First, activating inputs in the network are promiscuous, with broadly active morphogen mediators and transcriptional activators promoting the transcriptional programs of multiple progenitor domains (Figure 6) (Bailey et al., 2006; Bergsland et al., 2011; Oosterveen et al., 2012, 2013; Peterson et al., 2012). Second, specific cell identity is determined by a network of transcriptional repressors, which form a densely connected network, assuring that cells select a single definitive identity by repressing all inappropriate cell fates (Figures 2, 3, and 4) (Bailey et al., 2006; Lee and Pfaff, 2001; Lee et al., 2004; Muhr et al., 2001; Novershtern et al., 2011; Oosterveen et al., 2012, 2013; Peterson et al., 2012). Third, specification of identity requires not only repression of the “master regulator” TFs (NP-TFs) of other progenitor domains but also the direct repression of the “effector” genes expressed in other progenitor domains. Finally, the regulatory input into many target genes appears highly combinatorial and distributed over multiple CREs.

Activators Are Broad-Ranging and Promiscuous

In several developmental systems, transcriptional determinants have been identified that provide a tissue-specific platform for

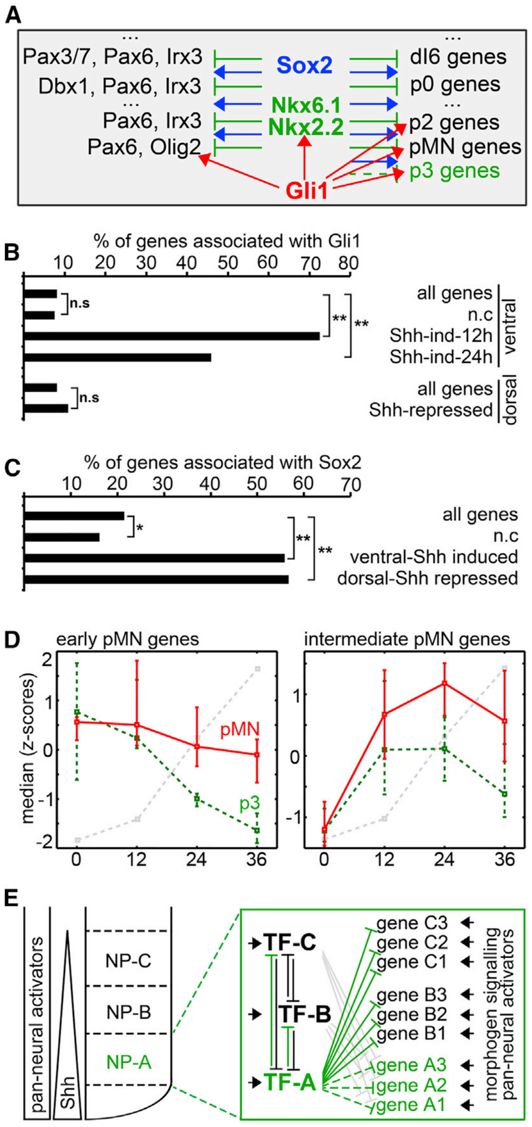

Figure 6. Shh via Gli and Sox2 Provide Direct Positive Input Into the Expression of Ventral Genes

(A) Experimental rationale: to test whether Shh directly provided the positive input into the transcription of ventral genes, Gli1 binding (Peterson et al., 2012) was analyzed with respect to genes induced and repressed by Shh signaling. To test whether the pan-neural TF Sox2 provided direct input into transcription of neural progenitor genes, Sox2 binding was analyzed with respect to genes expressed in ventral and dorsal domains.

(B) 73% of the genes upregulated by Shh at 12 hr and 46% of those upregulated at 24 hr in ventral progenitors (Figure S4A and Table S1, sheet S4) are associated with binding of Gli1. By contrast, few of the dorsal genes, repressed by Shh signaling, in p3 and/or pMN are associated with Gli1 binding. **p(χ^2) < 0.001, n.s, non-significant.

(C) Sox2 binds close to the majority of both ventral (induced by Shh in p3 or pMN at 12 or 24 hr) and dorsal (repressed by Shh in p3 or pMN at 24 hr) genes. **p(χ^2) < 0.001, *p(χ^2) < 0.005. See also Figures S4B and S4C.

(D) Median expression levels of early and intermediate pMN genes (genes expressed higher in pMN than p3, see Figure 2D) in pMN cells (red line) and p3 cells (green dotted line). The lines represent the median values of the indicated groups of genes and the error bars correspond to the 10th and 90th percentile values of each group. Note that the pMN genes are induced in both pMN and p3 cells simultaneously and they are only repressed in p3 later, at the time Nkx2.2 (gray dotted line) is activated at 24 hr. This suggests the common activatory inputs in p3 and pMN domains. 56% of intermediate genes are induced by Shh and 67% of them are bound by Gli1.

(E) Model summary. Four design features of the neural tube GRN. First, broadly expressed and promiscuous activating inputs from morphogen mediators and other transcriptional activators promote the transcriptional programs of multiple progenitor domains (A, B, C). Second, specific cell identity is determined by a network of transcriptional repressors (TF-A, TF-B, TF-C), these ensure cells select a single identity by repressing all inappropriate cell fates. Third, the repressors directly inhibit the expression of not only other repressors, but all the "effector" genes specific for other progenitor domains. This counteracts the direct positive inputs into all genes. Finally, the regulatory input into target genes is combinatorial and it is the integration of multiple, sometimes conflicting, inputs that determines cell identity.

the binding of signal mediators that activate cell type-specific gene expression programs (Heinz et al., 2010; Mullen et al., 2011; Trompouki et al., 2011). In neural progenitors, the combined binding of Sox TFs together with morphogen effectors have been proposed to activate expression of neural specific CREs and associated genes (Bailey et al., 2006; Oosterveen et al., 2012, 2013; Peterson et al., 2012; Wijgerde et al., 2002). We found that in addition to ventral NP-TFs, Sox2, and Gli1 (Peterson et al., 2012) were associated with most genes expressed in specific ventral domains. These included genes with "effector" functions, such as cell adhesion molecules and secreted factors. This suggests that Shh signaling together with broadly expressed activators directly activates the entire ventral progenitor programs (Bailey et al., 2006; Oosterveen et al., 2013). Sox2, but not Gli1, also bound to many of the genes characteristic of dorsal and intermediate progenitor domains, consistent with the idea that Sox2 provides a direct pan-neural activating input that regulates these genes in combination with other morphogen effectors (Bailey et al., 2006; Bergsland et al., 2011; Oosterveen et al., 2012, 2013). Thus, pan-neural TFs and morphogen effectors appear to directly activate the entire gene repertoire of neural progenitor domains by binding most genes expressed in specific progenitor subtypes. However, in contrast to predictions from in silico approaches (Oosterveen et al., 2013), we observe only partial overlap between Gli1 and Sox binding indicating that the two TFs might function through independent CREs to activate gene expression.

Both NP-TFs and Effector Genes Are Directly Repressed

The positioning of morphogen sources provides a spatial and temporal bias that influences neural tube patterning. Nevertheless, the precise arrangement and allocation of progenitor identity is dependent on the cross-repressive activity of NP-TFs expressed in adjacent domains (Balaskas et al., 2012; Briscoe et al., 2000; Cohen et al., 2014; Ericson et al., 1997; Sander et al., 2000; Vallstedt et al., 2001). Here we provide evidence that, in addition to cross-repressive activity between transcriptional determinants of adjacent progenitor domains, all genes corresponding to the alternative fate must be repressed (Figure 6E). Thus, to install the p3 program in progenitor cells, Nkx2.2 acting exclusively as a repressor (Figures 4A–4C) (Muhr et al., 2001), binds and inhibits genes encoding both NP-TFs and "effector" genes specific to alternative progenitor identities. This repressive activity is necessary to counter-balance the activatory function provided by broadly expressed activators and

mediators of morphogen signaling that bind to the same genes (Lee and Pfaff, 2001; Lee et al., 2004; Muhr et al., 2001; Oosterveen et al., 2012, 2013; Peterson et al., 2012). The segregation of repressor and activator functions negates the requirement for an NP-TF to act simultaneously as an activator for genes in its own domain and a repressor of adjacent domain genes.

The idea that NP-TFs act solely, or predominantly, as repressors is supported by the induction of a subset of pMN genes preceding the expression of Olig2, thus excluding a role for the pMN-defining NP-TF in activating these genes. Moreover, the pMN genes induced at early times are induced in both p3 and pMN cells and only later repressed in p3 by Nkx2.2, supporting the involvement of a common p3-pMN activators in their induction. Finally, Nkx2.2 and Olig2 bind and negatively regulate the expression of a subset of the genes expressed in their own domains. Although counter-intuitive, this suggests that NP-TFs decrease the expression of some of the genes with which they are coexpressed (see below).

The GRNs of other morphogen-patterned tissues appear to operate with similar principles. For example, to pattern the anterior-posterior axis of the *Drosophila* blastoderm, the graded TF Bicoid together with broadly expressed TF activators such as Zelda and STAT92E (Tsurumi et al., 2011; Xu et al., 2014) provide positive input to many target genes. Combinations of Gap-gene TFs selectively and directly repress subsets of these target genes to restrict expression to the appropriate domains (Chen et al., 2012; Surkova et al., 2008). Similarly along the dorsal-ventral axis of the blastoderm widely expressed activators and repressors, combined with domain-specific transcriptional determinants, are responsible for generating and positioning boundaries (Liang et al., 2012; Ozdemir et al., 2014; Reeves et al., 2012; Rushlow and Shvartsman, 2012). Hence combining broadly acting transcriptional determinants with a selective repressor-driven transcriptional network appears to be a common strategy for the allocation of cell identity in developing tissues.

Combinatorial and Direct Repression of Multiple Cell Identities

The presence of broadly expressed activators inducing multiple distinct progenitor programs, implies that specific NP-TFs must repress several inappropriate cellular identities. Accordingly, we provide evidence that the p3 determinants Nkx2.2 and Nkx6.1 directly repress not only the adjacent pMN identity but also non-adjacent intermediate/dorsal transcriptional programs. This prevents the indirect induction of any discordant gene expression that would otherwise result from repressive interactions solely between NP-TFs of adjacent domains. For example, Olig2 represses Irx3 to define the pMN/v2 boundary (Mizuguchi et al., 2001; Novitch et al., 2001) and, even though Nkx2.2 represses Olig2 to define the p3/pMN boundary (Novitch et al., 2001), Irx3 remains repressed in the p3 domain (Alaynick et al., 2011; Lovrics et al., 2014). Consistent with the direct repression of Irx3 by Nkx2.2 (Table S4), Irx3 expression expands ventrally in the Olig1/Olig2 mutant but does not cross the dorsal boundary of the p3 domain (Zhou and Anderson, 2002).

The NP-TFs expressed in a domain appear to act combinatorially to repress alternative fates. For example, ectopic expression of non-adjacent dorsally expressed NP-TFs, notably Dbx2 and Gsh1 (Gsx1), has been documented in the p3 domain of embryos lacking Nkx6.1 (Sander et al., 2000) despite the continued expression of Nkx2.2 in p3 cells. This indicates that Nkx6.1 alone or in combination with Nkx2.2, represses Dbx2 and Gsh1.

Conversely, genes specific for alternative fates are repressed independently in each domain by the combination of NP-TFs expressed in the specific progenitors. In the pMN domain, Olig2 appears to substitute for the repressor function of Nkx2.2; it binds and inhibits expression of many dorsal genes that are repressed in both pMN and p3 domains. Dbx2 and Gsh1 are not induced in pMN of Nkx6.1 mutants, suggesting Olig2-repressive activity alone is sufficient to block their expression (Sander et al., 2000). Similarly, analysis of a CRE associated with the Nkx6.1 gene, the activity of which is normally restricted to ventral p3-p2 domains, indicates it is independently repressed in the adjacent p1 domain by Dbx family members and in non-adjacent dorsal domains by members of the Msx family (Oosterveen et al., 2012). These factors bind to separate sites within the element (Oosterveen et al., 2012).

The combinatorial and independent nature of the gene-regulatory mechanism is emphasized by the observation that the NP-TFs bind and negatively regulate expression of many genes with which they are coexpressed. This seemingly paradoxical observation suggests that Boolean models that rely solely on the presence or absence of a repressor or activator will not be sufficient to fully describe developmental gene regulation. Instead the response of a gene must depend on the number and function of activators (both ubiquitous and morphogen regulated) and repressors (ubiquitous and NP-TFs), their respective arrangement and interactions within a CRE, as well as the number, function, and configuration of CREs associated with the gene. For example, Nkx2.2 binds close to and represses a substantial number of genes that are induced in p3 (Figures 5B and 5C). Nevertheless, when expressed in pMN cells, Nkx2.2 promotes the expression of these genes. This apparently contradictory result can be explained if the pMN determinants Olig2/Pax6 repress p3 genes more efficiently than Nkx2.2. Hence, the absence of Olig2 and Pax6 in p3 results in the induction of p3 genes by allowing activatory inputs to dominate the weaker repression provided by Nkx2.2. It is notable that Nkx2.2 and Olig2 appear to use different molecular mechanisms to repress gene expression: Nkx2.2 acts as Groucho-dependent repressor, but Olig2 lacks the Groucho-interacting domain (Lee and Pfaff, 2001). This difference, combined with the partially distinct subsets of CREs regulating these genes, could contribute to the difference in the target genes repressed in the two progenitor cell types.

In this view, the NP-TFs together with the positive inputs form a densely interconnected network (Novershtern et al., 2011) that determines the response of a gene. In progenitors in which a gene is expressed, the activatory inputs must dominate the negative inputs provided by the transcriptional repressors present in a cell. Each gene is likely to employ a different combination of strategies to overcome repression and a gene active in multiple domains might employ different strategies in each domain to escape repression by different sets of repressors. This is illustrated by the activity of two CREs associated with the Nkx6.1 gene (Figure S5). The gene is expressed broadly throughout the four most ventral domains, but the two enhancers

(−540 and −140 kb) are active only in distinct subsets of these domains depending on the nature of NP-TF bound to the particular element (Peterson et al., 2012). This mechanism, relying on gene-specific escape from repression (and therefore abrogating the need for domain-specific activation), has the potential to provide flexibility to the system. It might allow fine-tuning of the level of target gene expression and hence the possibility of generating more than one discrete level of expression (as observed for genes expressed in multiple progenitor domains). Mechanisms constructed with broad activation and densely connected networks of specific cross-repression readily produce the multistability necessary for stripes of gene expression. The non-contiguous stripes of reporter activity produced by Nkx6.1 –140-kb enhancer that integrates broad activatory inputs and domain-specific repression illustrates this (Figure S5). Moreover, the mechanism could offer a way to modify the GRN during the course of evolution (Wittkopp and Kalay, 2012) in order to interpret different combinations of extracellular signals or to introduce or eliminate specific cell identities.

Regulation Is Distributed over Multiple CREs

Despite the broad correlation between TF binding and CRE activity, single CREs rarely fully reflect the activity of a specific gene (Barolo, 2012; Levine, 2010). The identification of "shadow" enhancers (Hong et al., 2008) and data from chromatin interactome analyses suggest that multiple CREs often regulate expression of a single gene (Cannavò et al., 2016; Ghavi-Helm et al., 2014; Sanyal et al., 2012; Zhang et al., 2013). Analysis of the data from neural progenitors supports this view. Multiple distal CREs are found associated with most target genes. Moreover the inputs that control the expression of a gene in a specific progenitor type often appear to operate through distinct CREs. Thus, for example, Nkx6.1 which acts together with Nkx2.2 to repress genes discordant with p3 identity, shares only ~11% of its CREs with Nkx2.2 even though 61% of the genes associated with Nkx2.2 occupancy are also bound by Nkx6.1.

By contrast, Olig2 and Nkx2.2, which are not expressed in the same progenitor types, share a substantial number of CREs. This is especially evident with dorsal genes repressed in both p3 and pMN domains; in these cases 53% of Nkx2.2 binding coincided with Olig2 binding. Thus, Nkx2.2 and Olig2 use a set of common CREs to maintain the exclusion of inappropriate gene expression. Hence during the progressive establishment of pattern in the ventral neural tube (Dessaud et al., 2007; Jeong and McMahon, 2005), the induction of Nkx2.2, which represses Olig2, will be accompanied by the replacement of Nkx2.2 on CREs previously occupied by Olig2. The two TFs favor distinct DNA-binding motifs (Figure S1B), and in the majority of shared CREs these motifs are not in close proximity. Thus, the two TFs would, in principle, be able to bind simultaneously to the same CREs and thus ensure the continued repression of inappropriate gene expression during the transition in cell identity.

The NP-GRN and Interpretation of Signaling Gradients

The independent repression of progenitor-specific transcriptional programs by multiple NP-TFs specific for alternative fates is consistent with the instructive role of the transcriptional network in establishing the differential response of genes to morphogen signaling (Balaskas et al., 2012; Cohen et al., 2014). In this model, it is the combinatorial action of NP-TF repressors present in a cell at a given time that determines the spatial-temporal response of ventral target genes to Shh. This mechanism also provides an explanation for how cells interpret the temporally changing levels of Gli activity to produce the observed dynamics of neural tube patterning (Balaskas et al., 2012; Cohen et al., 2014). Initially, Shh blocks the processing of Gli3 into its repressor form, thus removing this repressive activity from ventral genes. Later it provides direct positive input to overcome the repressive activity of NP-TFs. Consequently, in the case of some genes, such as Ptch1, Nkx6.1, and Nkx6.2, the removal of the repressor form of Gli3 is sufficient and these genes are induced rapidly and by low levels of Shh (Litingtung and Chiang, 2000; Persson et al., 2002; Wijgerde et al., 2002). The absence of Gli repressor in the CREs of these genes is presumably sufficient to allow Sox2 and perhaps other pan-neural transactivators to induce the expression (Oosterveen et al., 2012). The dorsal limits of Nkx6.1 and Nkx6.2 are restricted to p1 and p2, respectively, by repression from Dbx and Msx NP-TFs in the intermediate and dorsal neural tube (Oosterveen et al., 2012; Vallstedt et al., 2001). Olig2, which has to overcome repression from both Gli3R and Irx3 (Novitch et al., 2001; Persson et al., 2002; Sasai et al., 2014), is induced later and restricted to more ventral domains receiving higher morphogen activity. Olig2 induction is then followed by Nkx2.2. In the case of Nkx2.2, higher levels and longer durations of Shh signaling are required to produce sufficient Gli activator to overcome the repression by Irx3, Pax6, and Olig2 (which replaces Irx3) (Oosterveen et al., 2012; Balaskas et al., 2012; Jeong and McMahon, 2005). A similar rationale could explain the differential timing of induction of the non-NP-TF pMN genes (Figure 2D): the genes induced by Shh at 12 hr are activated following the reduction in Gli3R, whereas genes induced at 24 hr require induction of Olig2 to repress Irx3.

Taken together, our findings shed light on the molecular mechanism and design features of the transcriptional network that establishes the pattern in the vertebrate neural tube. The regulatory links between the repressors in the transcriptional network provide a mechanism to interpret the dynamic morphogen input and select the appropriate transcriptional identity for the position along the patterning axes (Balaskas et al., 2012; Chen et al., 2012; Cohen et al., 2014; Manu et al., 2009). Given the similarity in the operating principles of this system with other developmental systems, this suggests a general architecture for morphogen-controlled GRNs that is likely to be relevant for other tissues.

EXPERIMENTAL PROCEDURES

Cell Lines and Neural Progenitor Differentiation

The HPRT locus of ES^{tet-ON} cell line was targeted with Tet-responsive transgenes allowing inducible expression of mouse Nkx2.2, Nkx6.1, Nkx2.2-2A-Nkx6.1, Nkx6.1-2A-Nkx2.2, Nkx2.2HD-EnR, and Olig2 cDNAs as described (Gouti and Gavalas, 2008; Selfridge et al., 1992). ES^{tet-ON}-derived cell lines were maintained on feeders in leukemia inhibitory factor-supplemented medium, containing 15% fetal calf serum. Sox1-GFP ESCs (Ying et al., 2003) (a gift from A. Smith) were maintained feeder free on gelatin-coated dishes, in the same medium. The monolayer differentiation is modified from Andersson et al. (2006) and Ying et al. (2003) and described in Sasai et al. (2014). For dorsal (30RA) or dorsal (300RA) differentiation, 30 or 300 nM RA (Sigma R2526), respectively, was added to N2B27 medium at day 3. For pMN differentiation, in addition to 300 nM RA, 1 μg/ml recombinant Shh was added from day 3.5.

For p3 differentiation, in addition to 30 nM RA, 2 μg/ml recombinant Shh was added from day 3.5. FP progenitors were generated by adding 2 μg/ml Shh to the N2B27 medium from day 3.5. To induce the expression of NP-TF-Rs, 1 μg/ml doxycycline (Sigma) was added to medium as described in the text. From day 3, the medium was replaced every 12 hr.

In Ovo Chick Electroporation

In ovo chick electroporation was performed as described. RCAS-Nkx2.2 construct (Briscoe et al., 2000) was used for ventral electroporation of Nkx2.2. For lateral electroporation of Foxa2 or Foxa2 and Nkx2.2, pCAGGS expression constructs with full-length mouse Foxa2 (Sasai et al., 2014) and full length chick Nkx2.2 (Muhr et al., 2001) proteins were used. All animal experiments were performed under a UK Home Office project license (PPL80/2528) within the conditions of the Animals (Scientific Procedures) Act 1986 and approved by the Animal Welfare and Ethical Review Panel of the MRC-National Institute for Medical Research.

Immunohistochemistry

Immunohistochemistry on neural progenitors and chick sections was performed as described (Sasai et al., 2014). For the list of antibodies, see Supplemental Experimental Procedures.

ChIP-Seq

ChIP was performed as described (Kutejova et al., 2008). Briefly, 1–3 × 10^8 neural progenitor cells (derived from Sox1-GFP line) were crosslinked for 23 min at 4°C with 1% formaldehyde at day 5 (36 hr) of differentiation. Chromatin was sonicated using a Bioruptor (Diagenode) to 200- to 500-bp fragments and incubated with 6 μg of rabbit anti-Nkx2.2, rabbit anti-Nkx6.1 (this manuscript), rabbit anti-Olig2 (Millipore AB9610), or goat anti-Sox2 (Santa Cruz sc-17320X) antibodies per 2 × 10^7 cells, overnight. Immunoprecipitated chromatin fragments were purified using protein A or G-coupled Dynabeads (Life Technologies). The libraries were prepared using standard Illumina protocols and sequenced on a GAIIx Illumina platform (GeneCore, EMBL). Following sequencing, 36-bp single-end reads were aligned to GRCm38 genome assembly using Bowtie (Langmead et al., 2009). MACS (Zhang et al., 2008) was used to call peaks. Peaks were associated with the closest genes using CisGenome (Ji et al., 2008). De novo motif search was performed using Homer (Heinz et al., 2010) and TomTom (Gupta et al., 2007) was used to search for similar motifs in known datasets. For additional information, see Supplemental Experimental Procedures and Table S4. The accession number for the raw sequence data reported in this paper is ENA: PRJEB7682 (Table S2).

RNA-Seq

Neural progenitors were lysed in Trizol (Life Technologies) at times indicated in the text and total RNA was purified using RNeasy purification kit (Life Technologies). The libraries were prepared using Illumina's TruSeq RNA Sample Preparation Kit v2 and sequenced on Illumina HiSeq 2000. Paired-end reads were aligned to GRCm38 genome using TopHat (Trapnell et al., 2009), and the number of reads per feature in Ensembl Genes 77 GTF table were counted using HTSeq (Anders et al., 2015). The pairwise differential expression analysis was performed using DESeq (Anders and Huber, 2010). For additional information, see Supplemental Experimental Procedures and Tables S1, S3, and S5, which contain details of gene lists and replicate samples used to generate specific figure panels. The accession number for the raw sequence data reported in this paper is ENA: PRJEB7682 (Table S2)

ACCESSION NUMBERS

The accession number for the raw sequence data reported in this paper is ENA: PRJEB7682.

SUPPLEMENTAL INFORMATION

Supplemental Information includes Supplemental Experimental Procedures, five figures, and five tables and can be found with this article online at http://dx.doi.org/10.1016/j.devcel.2016.02.013.

AUTHOR CONTRIBUTIONS

E.K., N.S., and A.S. performed the experiments. E.K. analyzed the data. E.K. and J.B. conceived the work and wrote the manuscript. M.G. developed the doxycycline-inducible ESC system.

ACKNOWLEDGMENTS

We thank Dominic van Essen, Francois Guillemot, Vanessa Ribes, J.P. Vincent, and members of the laboratory for comments on the manuscript; Andy McMahon and Yuichi Nishi for communicating results prior to publication; Jens Kleinjung for discussions and support; Vangelis Christodoulou for help with purification of recombinant proteins; Fabrice Prin for help with chick electroporations; Abdul Sesay, Leena Bhaw-Rosun, Harsha Jani, and Deborah Jackson for help with high-throughput mRNA sequencing; and Thomas M. Jessell, Jamel Chelly, Ole Madsen, and BCBC Antibody Core for antibodies. This work was funded by the MRC (U117560541) and Wellcome Trust (WT098326MA). E.K. was supported by EMBO ALTF 1086–2008.

Received: November 5, 2015
Revised: December 29, 2015
Accepted: February 12, 2016
Published: March 10, 2016

REFERENCES

Alaynick, W.A., Jessell, T.M., and Pfaff, S.L. (2011). SnapShot: spinal cord development. Cell *146*, 178–178.e1.

Anders, S., and Huber, W. (2010). Differential expression analysis for sequence count data. Genome Biol. *11*, R106.

Anders, S., Pyl, P.T., and Huber, W. (2015). HTSeq-a Python framework to work with high-throughput sequencing data. Bioinformatics *31*, 166–169.

Andersson, E., Tryggvason, U., Deng, Q., Friling, S., Alekseenko, Z., Robert, B., Perlmann, T., and Ericson, J. (2006). Identification of intrinsic determinants of midbrain dopamine neurons. Cell *124*, 393–405.

Bailey, P.J., Klos, J.M., Andersson, E., Karlen, M., Källström, M., Ponjavic, J., Muhr, J., Lenhard, B., Sandelin, A., and Ericson, J. (2006). A global genomic transcriptional code associated with CNS-expressed genes. Exp. Cell Res. *312*, 3108–3119.

Balaskas, N., Ribeiro, A., Panovska, J., Dessaud, E., Sasai, N., Page, K.M., Briscoe, J., and Ribes, V. (2012). Gene regulatory logic for reading the Sonic Hedgehog signaling gradient in the vertebrate neural tube. Cell *148*, 273–284.

Barolo, S. (2012). Shadow enhancers: frequently asked questions about distributed cis-regulatory information and enhancer redundancy. Bioessays *34*, 135–141.

Bergsland, M., Ramsköld, D., Zaouter, C., Klum, S., Sandberg, R., and Muhr, J. (2011). Sequentially acting Sox transcription factors in neural lineage development. Genes Dev. *25*, 2453–2464.

Briscoe, J., Sussel, L., Serup, P., Hartigan-O'Connor, D., Jessell, T.M., Rubenstein, J.L., and Ericson, J. (1999). Homeobox gene Nkx2.2 and specification of neuronal identity by graded Sonic hedgehog signalling. Nature *398*, 622–627.

Briscoe, J., Pierani, A., Jessell, T.M., and Ericson, J. (2000). A homeodomain protein code specifies progenitor cell identity and neuronal fate in the ventral neural tube. Cell *101*, 435–445.

Briscoe, J., Chen, Y., Jessell, T.M., and Struhl, G. (2001). A hedgehog-insensitive form of patched provides evidence for direct long-range morphogen activity of sonic hedgehog in the neural tube. Mol. Cell *7*, 1279–1291.

Cannavò, E., Khoueiry, P., Garfield, D.A., Geeleher, P., Zichner, T., Gustafson, E.H., Ciglar, L., Korbel, J.O., and Furlong, E.E. (2016). Shadow enhancers are pervasive features of developmental regulatory networks. Curr. Biol. *26*, 38–51.

Chen, H., Xu, Z., Mei, C., Yu, D., and Small, S. (2012). A system of repressor gradients spatially organizes the boundaries of bicoid-dependent target genes. Cell *149*, 618–629.

Cohen, M., Page, K.M., Perez-Carrasco, R., Barnes, C.P., and Briscoe, J. (2014). A theoretical framework for the regulation of Shh morphogen-controlled gene expression. Development *141*, 3868–3878.

Davidson, E.H. (2010). Emerging properties of animal gene regulatory networks. Nature *468*, 911–920.

Dessaud, E., Yang, L.L., Hill, K., Cox, B., Ulloa, F., Ribeiro, A., Mynett, A., Novitch, B.G., and Briscoe, J. (2007). Interpretation of the sonic hedgehog morphogen gradient by a temporal adaptation mechanism. Nature *450*, 717–720.

Dessaud, E., McMahon, A.P., and Briscoe, J. (2008). Pattern formation in the vertebrate neural tube: a sonic hedgehog morphogen-regulated transcriptional network. Development *135*, 2489–2503.

Ericson, J., Rashbass, P., Schedl, A., Brenner-Morton, S., Kawakami, A., van Heyningen, V., Jessell, T.M., and Briscoe, J. (1997). Pax6 controls progenitor cell identity and neuronal fate in response to graded Shh signaling. Cell *90*, 169–180.

Ghavi-Helm, Y., Klein, F.A., Pakozdi, T., Ciglar, L., Noordermeer, D., Huber, W., and Furlong, E.E.M. (2014). Enhancer loops appear stable during development and are associated with paused polymerase. Nature *512*, 96–100.

Gouti, M., and Gavalas, A. (2008). Hoxb1 controls cell fate specification and proliferative capacity of neural stem and progenitor cells. Stem Cells *26*, 1985–1997.

Gupta, S., Stamatoyannopoulos, J.A., and Bailey, T.L. (2007). Quantifying similarity between motifs. Genome Biol. *8*, R24.

Heinz, S., Benner, C., Spann, N., Bertolino, E., Lin, Y.C., Laslo, P., Cheng, J.X., Murre, C., Singh, H., and Glass, C.K. (2010). Simple combinations of lineage-determining transcription factors prime cis-regulatory elements required for macrophage and B cell identities. Mol. Cell *38*, 576–589.

Hong, J.-W., Hendrix, D.A., and Levine, M.S. (2008). Shadow enhancers as a source of evolutionary novelty. Science *321*, 1314.

Imayoshi, I., Isomura, A., Harima, Y., Kawaguchi, K., Kori, H., Miyachi, H., Fujiwara, T., Ishidate, F., and Kageyama, R. (2013). Oscillatory control of factors determining multipotency and fate in mouse neural progenitors. Science *342*, 1203–1208.

Jeong, J., and McMahon, A.P. (2005). Growth and pattern of the mammalian neural tube are governed by partially overlapping feedback activities of the hedgehog antagonists patched 1 and Hhip1. Development *132*, 143–154.

Jessell, T.M. (2000). Neuronal specification in the spinal cord: inductive signals and transcriptional codes. Nat. Rev. Genet. *1*, 20–29.

Ji, H., Jiang, H., Ma, W., Johnson, D.S., Myers, R.M., and Wong, W.H. (2008). An integrated software system for analyzing ChIP-chip and ChIP-seq data. Nat. Biotechnol. *26*, 1293–1300.

Kicheva, A., Bollenbach, T., Ribeiro, A., Valle, H.P., Lovell-Badge, R., Episkopou, V., and Briscoe, J. (2014). Coordination of progenitor specification and growth in mouse and chick spinal cord. Science *345*, 1254927.

Kutejova, E., Engist, B., Self, M., Oliver, G., Kirilenko, P., and Bobola, N. (2008). Six2 functions redundantly immediately downstream of Hoxa2. Development *135*, 1463–1470.

Langmead, B., Trapnell, C., Pop, M., and Salzberg, S.L. (2009). Ultrafast and memory-efficient alignment of short DNA sequences to the human genome. Genome Biol. *10*, R25.

Lee, S.K., and Pfaff, S.L. (2001). Transcriptional networks regulating neuronal identity in the developing spinal cord. Nat. Neurosci. *4* (*Suppl*), 1183–1191.

Lee, S.-K., Jurata, L.W., Funahashi, J., Ruiz, E.C., and Pfaff, S.L. (2004). Analysis of embryonic motoneuron gene regulation: derepression of general activators function in concert with enhancer factors. Development *131*, 3295–3306.

Lek, M., Dias, J.M., Marklund, U., Uhde, C.W., Kurdija, S., Lei, Q., Sussel, L., Rubenstein, J.L., Matise, M.P., Arnold, H.-H., et al. (2010). A homeodomain feedback circuit underlies step-function interpretation of a Shh morphogen gradient during ventral neural patterning. Development *137*, 4051–4060.

Levine, M. (2010). Transcriptional enhancers in animal development and evolution. Curr. Biol. *20*, R754–R763.

Liang, H.L., Xu, M., Chuang, Y.C., and Rushlow, C. (2012). Response to the BMP gradient requires highly combinatorial inputs from multiple patterning systems in the Drosophila embryo. Development *139*, 1956–1964.

Litingtung, Y., and Chiang, C. (2000). Specification of ventral neuron types is mediated by an antagonistic interaction between Shh and Gli3. Nat. Neurosci. *3*, 979–985.

Lovrics, A., Gao, Y., Juhász, B., Bock, I., Byrne, H.M., Dinnyés, A., and Kovács, K.A. (2014). Boolean modelling reveals new regulatory connections between transcription factors orchestrating the development of the ventral spinal cord. PLoS One *9*, e111430.

Mansour, A.A., Khazanov-Zisman, S., Netser, Y., Klar, A., and Ben-Arie, N. (2014). Nato3 plays an integral role in dorsoventral patterning of the spinal cord by segregating floor plate/p3 fates via Nkx2.2 suppression and Foxa2 maintenance. Development *141*, 574–584.

Manu, Surkova, S., Spirov, A.V., Gursky, V.V., Janssens, H., Kim, A.-R., Radulescu, O., Vanario-Alonso, C.E., Sharp, D.H., Samsonova, M., et al. (2009). Canalization of gene expression in the Drosophila blastoderm by gap gene cross regulation. PLoS Biol. *7*, e1000049.

Mizuguchi, R., Sugimori, M., Takebayashi, H., Kosako, H., Nagao, M., Yoshida, S., Nabeshima, Y., Shimamura, K., and Nakafuku, M. (2001). Combinatorial roles of olig2 and neurogenin2 in the coordinated induction of pan-neuronal and subtype-specific properties of motoneurons. Neuron *31*, 757–771.

Muhr, J., Andersson, E., Persson, M., Jessell, T.M., and Ericson, J. (2001). Groucho-mediated transcriptional repression establishes progenitor cell pattern and neuronal fate in the ventral neural tube. Cell *104*, 861–873.

Mullen, A.C., Orlando, D.A., Newman, J.J., Lovén, J., Kumar, R.M., Bilodeau, S., Reddy, J., Guenther, M.G., DeKoter, R.P., and Young, R.A. (2011). Master transcription factors determine cell-type-specific responses to TGF-β signaling. Cell *147*, 565–576.

Nishi, Y., Zhang, X., Jeong, J., Peterson, K.A., Vedenko, A., Bulyk, M.L., Hide, W.A., and McMahon, A.P. (2015). A direct fate exclusion mechanism by Sonic hedgehog-regulated transcriptional repressors. Development *142*, 3286–3293.

Novershtern, N., Subramanian, A., Lawton, L.N., Mak, R.H., Haining, W.N., McConkey, M.E., Habib, N., Yosef, N., Chang, C.Y., Shay, T., et al. (2011). Densely interconnected transcriptional circuits control cell states in human hematopoiesis. Cell *144*, 296–309.

Novitch, B.G., Chen, A.I., and Jessell, T.M. (2001). Coordinate regulation of motor neuron subtype identity and pan-neuronal properties by the bHLH repressor Olig2. Neuron *31*, 773–789.

Novitch, B.G., Wichterle, H., Jessell, T.M., and Sockanathan, S. (2003). A requirement for retinoic acid-mediated transcriptional activation in ventral neural patterning and motor neuron specification. Neuron *40*, 81–95.

Oosterveen, T., Kurdija, S., Alekseenko, Z., Uhde, C.W., Bergsland, M., Sandberg, M., Andersson, E., Dias, J.M., Muhr, J., and Ericson, J. (2012). Mechanistic differences in the transcriptional interpretation of local and long-range Shh morphogen signaling. Dev. Cell *23*, 1006–1019.

Oosterveen, T., Kurdija, S., Ensterö, M., Uhde, C.W., Bergsland, M., Sandberg, M., Sandberg, R., Muhr, J., and Ericson, J. (2013). SoxB1-driven transcriptional network underlies neural-specific interpretation of morphogen signals. Proc. Natl. Acad. Sci. USA *110*, 7330–7335.

Ozdemir, A., Ma, L., White, K.P., and Stathopoulos, A. (2014). Su(H)-mediated repression positions gene boundaries along the dorsal-ventral axis of drosophila embryos. Dev. Cell *31*, 100–113.

Persson, M., Stamataki, D., Welscher te, P., Andersson, E., Böse, J., Rüther, U., Ericson, J., and Briscoe, J. (2002). Dorsal-ventral patterning of the spinal cord requires Gli3 transcriptional repressor activity. Genes Dev. *16*, 2865–2878.

Peterson, K.A., Nishi, Y., Ma, W., Vedenko, A., Shokri, L., Zhang, X., McFarlane, M., Baizabal, J.M., Junker, J.P., van Oudenaarden, A., et al. (2012). Neural-specific Sox2 input and differential Gli-binding affinity provide context and positional information in Shh-directed neural patterning. Genes Dev. *26*, 2802–2816.

Reeves, G.T., Trisnadi, N., Truong, T.V., Nahmad, M., Katz, S., and Stathopoulos, A. (2012). Dorsal-ventral gene expression in the Drosophila embryo reflects the dynamics and precision of the dorsal nuclear gradient. Dev. Cell *22*, 544–557.

Ribes, V., Balaskas, N., Sasai, N., Cruz, C., Dessaud, E., Cayuso, J., Tozer, S., Yang, L.L., Novitch, B., Martí, E., et al. (2010). Distinct Sonic Hedgehog signaling dynamics specify floor plate and ventral neuronal progenitors in the vertebrate neural tube. Genes Dev. *24*, 1186–1200.

Rushlow, C.A., and Shvartsman, S.Y. (2012). Temporal dynamics, spatial range, and transcriptional interpretation of the Dorsal morphogen gradient. Curr. Opin. Genet. Dev. *22*, 542–546.

Sander, M., Paydar, S., Ericson, J., Briscoe, J., Berber, E., German, M., Jessell, T.M., and Rubenstein, J.L. (2000). Ventral neural patterning by Nkx homeobox genes: Nkx6.1 controls somatic motor neuron and ventral interneuron fates. Genes Dev. *14*, 2134–2139.

Sanyal, A., Lajoie, B.R., Jain, G., and Dekker, J. (2012). The long-range interaction landscape of gene promoters. Nature *489*, 109–113.

Sasai, N., Kutejova, E., and Briscoe, J. (2014). Integration of signals along orthogonal axes of the vertebrate neural tube controls progenitor competence and increases cell diversity. PLoS Biol. *12*, e1001907.

Selfridge, J., Pow, A.M., McWhir, J., Magin, T.M., and Melton, D.W. (1992). Gene targeting using a mouse HPRT minigene/HPRT-deficient embryonic stem cell system: inactivation of the mouse ERCC-1 gene. Somat. Cell Mol. Genet. *18*, 325–336.

Surkova, S., Kosman, D., Kozlov, K., Manu, Myasnikova, E., Samsonova, A.A., Spirov, A., Vanario-Alonso, C.E., Samsonova, M., and Reinitz, J. (2008). Characterization of the Drosophila segment determination morphome. Dev. Biol. *313*, 844–862.

Trapnell, C., Pachter, L., and Salzberg, S.L. (2009). TopHat: discovering splice junctions with RNA-Seq. Bioinformatics *25*, 1105–1111.

Trompouki, E., Bowman, T.V., Lawton, L.N., Fan, Z.P., Wu, D.-C., DiBiase, A., Martin, C.S., Cech, J.N., Sessa, A.K., Leblanc, J.L., et al. (2011). Lineage regulators direct BMP and Wnt pathways to cell-specific programs during differentiation and regeneration. Cell *147*, 577–589.

Tsurumi, A., Xia, F., Li, J., Larson, K., LaFrance, R., and Li, W.X. (2011). STAT is an essential activator of the zygotic genome in the early Drosophila embryo. PLoS Genet. *7*, e1002086.

Vallstedt, A., Muhr, J., Pattyn, A., Pierani, A., Mendelsohn, M., Sander, M., Jessell, T.M., and Ericson, J. (2001). Different levels of repressor activity assign redundant and specific roles to Nkx6 genes in motor neuron and interneuron specification. Neuron *31*, 743–755.

Vokes, S.A., Ji, H., McCuine, S., Tenzen, T., Giles, S., Zhong, S., Longabaugh, W.J.R., Davidson, E.H., Wong, W.H., and McMahon, A.P. (2007). Genomic characterization of Gli-activator targets in sonic hedgehog-mediated neural patterning. Development *134*, 1977–1989.

Wijgerde, M., McMahon, J.A., Rule, M., and McMahon, A.P. (2002). A direct requirement for Hedgehog signaling for normal specification of all ventral progenitor domains in the presumptive mammalian spinal cord. Genes Dev. *16*, 2849–2864.

Wittkopp, P.J., and Kalay, G. (2012). Cis-regulatory elements: molecular mechanisms and evolutionary processes underlying divergence. Nat. Rev. Genet. *13*, 59–69.

Xu, Z., Chen, H., Ling, J., Yu, D., Struffi, P., and Small, S. (2014). Impacts of the ubiquitous factor Zelda on Bicoid-dependent DNA binding and transcription in Drosophila. Genes Dev. *28*, 608–621.

Ying, Q.-L., Stavridis, M., Griffiths, D., Li, M., and Smith, A. (2003). Conversion of embryonic stem cells into neuroectodermal precursors in adherent monoculture. Nat. Biotechnol. *21*, 183–186.

Zhang, Y., Liu, T., Meyer, C.A., Eeckhoute, J., Johnson, D.S., Bernstein, B.E., Nusbaum, C., Myers, R.M., Brown, M., Li, W., et al. (2008). Model-based analysis of ChIP-Seq (MACS). Genome Biol. *9*, R137.

Zhang, Y., Wong, C.-H., Birnbaum, R.Y., Li, G., Favaro, R., Ngan, C.Y., Lim, J., Tai, E., Poh, H.M., Wong, E., et al. (2013). Chromatin connectivity maps reveal dynamic promoter–enhancer long-range associations. Nature *504*, 306–310.

Zhou, Q., and Anderson, D.J. (2002). The bHLH transcription factors OLIG2 and OLIG1 couple neuronal and glial subtype specification. Cell *109*, 61–73.

Zhou, Q., Choi, G., and Anderson, D.J. (2001). The bHLH transcription factor Olig2 promotes oligodendrocyte differentiation in collaboration with Nkx2.2. Neuron *31*, 791–807.

Developmental Cell, Volume *36*

Supplemental Information

Neural Progenitors Adopt Specific Identities by Directly Repressing All Alternative Progenitor Transcriptional Programs

Eva Kutejova, Noriaki Sasai, Ankita Shah, Mina Gouti, and James Briscoe

Inventory of Supplemental Materials

Supplemental Figures and Legends

Figure S1. Gene ontology, binding motifs. Related to Figure 1.

Figure S2. Inducible ES cell lines. Related to Figures 2, 3, 4 and 5.

Figure S3. Identification of genes specific for definitive FP – "late" FP genes. Related to Figure 2 and Table S1, sheet S3.

Figure S4. Expression kinetics of genes induced by Shh. Shh-regulated genes associate with activator and repressor peaks. Related to Figure 6 and Table S1, sheet S4.

Figure S5. Activity of individual ventral CREs correlates with direct positive input of Sox2/3 and Gli1 and direct negative inputs of Nkx2.2, Olig2 and Nkx6.1. Related to Figures 5 and 6.

Supplemental Tables (available as separate files)

Table S1. Genes, their expression and classification. Related to Figures 1D, 2B, 2F, 3B, 3F, 3G, S3B-C and S4A.

Table S2. Lists of RNA and ChIP-seq samples, accession numbers. Related to Figures 1, 2, 3, 4, 5, 6, S1, S3, S4 and S5.

Table S3. RNA-seq: read counts. Related to Figures 1, 2, 3, 4, 5, 6, S1, S3 and S4.

Table S4. ChIP-seq: peaks. Related to Figures 1, 2, 3, 5, 6, S1, S4 and S5.

Table S5. Gene lists and samples used to generate heatmaps and boxplots. Related to Figures 1, 2, 3, 4, 5, 6, S1, S3 and S4.

Supplemental Experimental Procedures

Antibodies for immunohistochemistry.

Production of recombinant C25II-Shh protein.

Production of anti-Nkx2.2 and anti-Nkx6.1 antibodies.

ChIP-seq analysis.

RNA-seq analysis. Gene lists. Heatmaps. Boxplots.

Supplemental References

Supplemental Figures and Legends

Figure S1

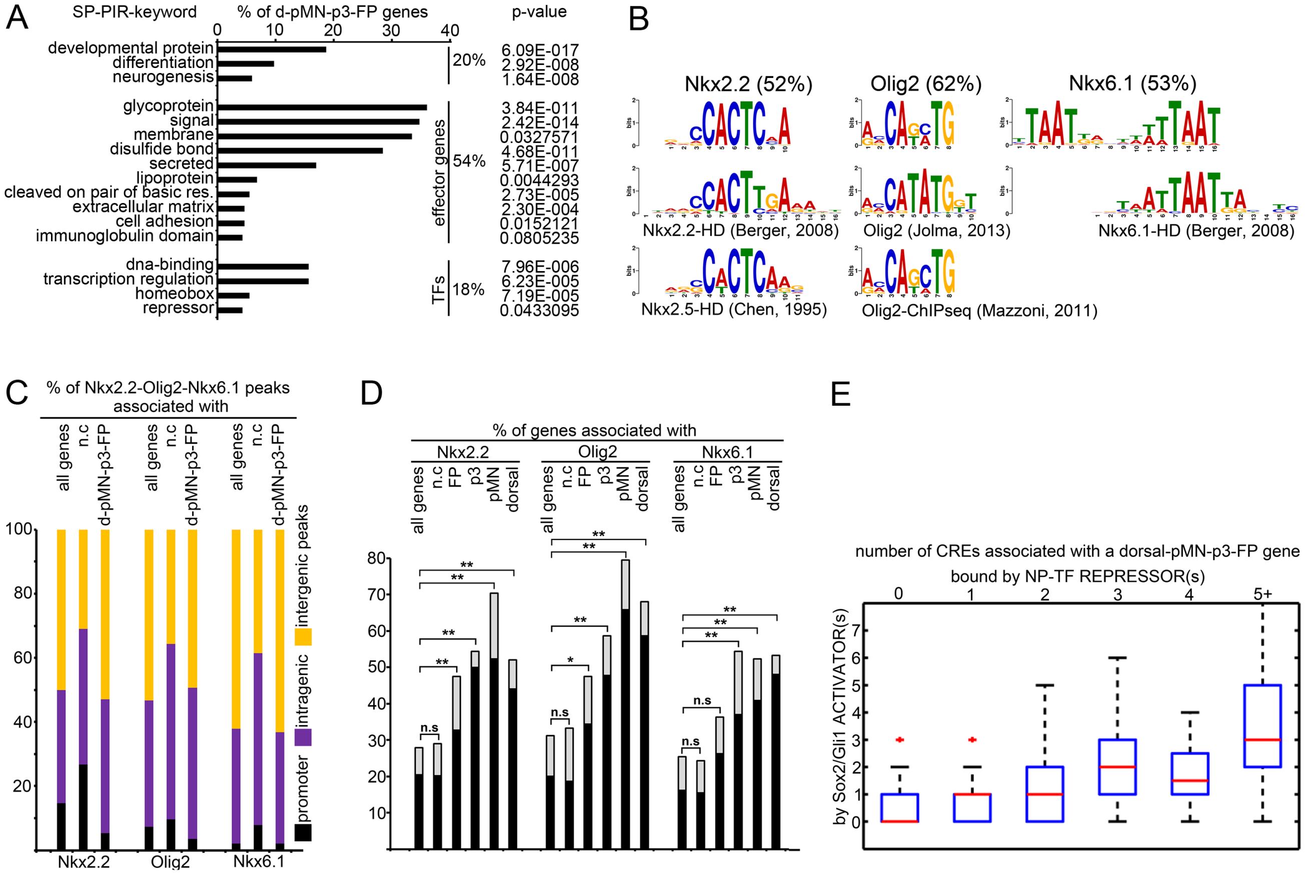

Figure S1. Gene ontology, binding motifs. Related to Figure 1.

A. Gene ontology analysis indicates that genes differentially expressed in FP-p3-pMN-dorsal (Fig. 1D) are enriched in TFs (18%) and membrane-associated/secreted molecules (54%) compared to all genes, p<0.1. The analysis was performed using DAVID (Huang et al., 2009) and is based on Swiss-Prot (SP) and Protein Information Resource (PIR) Keywords (SP_PIR_KEYWORD).

B. De novo motifs searches from regions bound by Nkx2.2, Olig2, Nkx6.1 recovered the corresponding *in vitro* defined motifs (Berger et al., 2008; Chen and Schwartz, 1995; Jolma et al., 2013) and motifs recovered from other ChIP-seq datasets (Olig2) (Mazzoni et al., 2011).

C. Nkx2.2, Olig2 and Nkx6.1 bind to intragenic and intergenic regions rather than promoters. Based on their genomic position with respect to the RefSeq transcripts, peaks were first classified into promoter (TSS-3kb to TSS, black), intragenic (TSS to TES, yellow, more than 70% intronic) and intergenic (other, purple) peaks and then associated with the closest genes. All three TFs were depleted from the promoter regions, especially from the promoters of the regulated genes - genes differentially expressed in dorsal-pMN-p3-FP (Fig. 1D), (black).

D. The proportion of dorsal-pMN-p3-FP genes associated with Nkx2.2-Olig2-Nkx6.1 further increases when two closest genes on each side of the peak are analysed. Compare black bars (one closest gene, Fig. 1E) with grey bars (two closest genes). The enrichment compared to all genes is highly significant, especially when the promoter peaks of Nkx2.2 (that do not seem to confer regulatory properties, Fig. S1C) are excluded (shown here for two closest genes). ** $p(X^2)<0.001$, * $p(X^2)<0.025$.

E. Dorsal-pMN-p3-FP genes that lack a CRE bound by NP-TFs in their immediate vicinity also tend to lack binding of activator TFs Sox2 and Gli1. The box plot indicates the number of Sox2 and/or Gli1 bound CREs compared to number of NP-TF bound CREs for each regulated gene. This agrees with the observation that the regulatory modules associated with a gene or a set of genes tend to cluster. D and E suggest that the regulated genes not harbouring NP-TF bound CREs in their immediate vicinity are likely to be controlled by CREs separated by one or more genes.

Figure S2

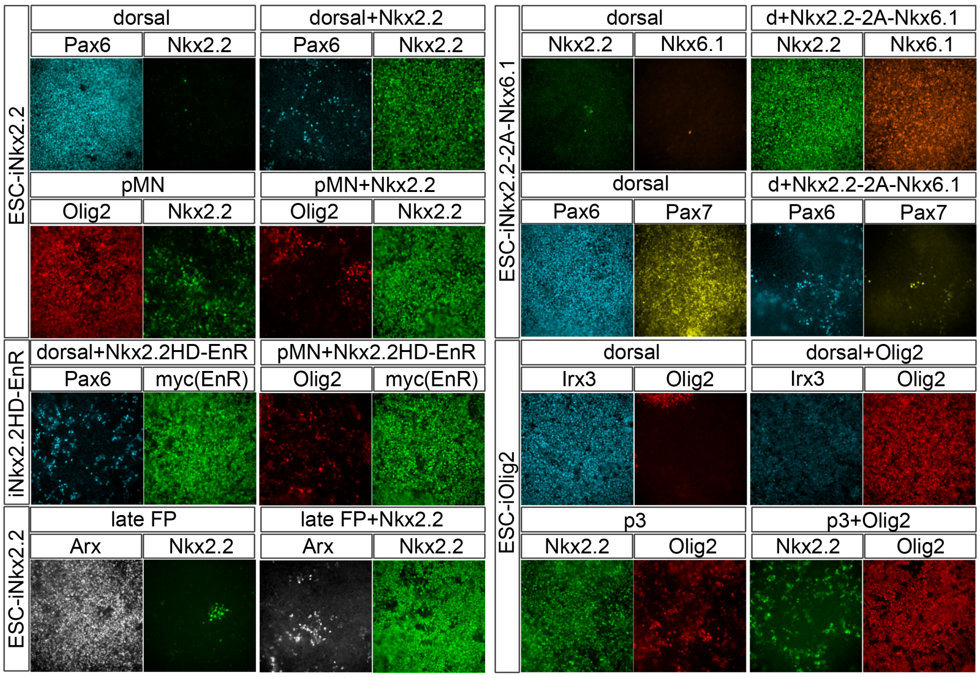

Figure S2. Inducible ES cell lines. Related to Figures 2, 3, 4 and 5.

Expression of Nkx2.2 (ESC-iNkx2.2 cell line) in dorsal, pMN and FP cells leads to the downregulation of the corresponding TFs Pax6, Olig2 and Arx. Similarly, overexpression of Nkx2.2HD-EnR using ESC-iNkx2.2HD-EnR cell line in dorsal and pMN abolishes expression of Pax6 and Olig2. Expression of Nkx2.2 and Nkx6.1 (ESC-iNkx2.2-2A-Nkx6.1) in dorsal cells downregulates Pax6 and Pax7. Expression of Olig2 (iNPC-Olig2) in dorsal and p3 cells represses Irx3 and Nkx2.2 respectively. Expression of marker genes in all conditions was analysed by immunofluorescence at day 5 (36h) except for the late FP, where the pictures were taken at day 6 (60h).

Figure S3

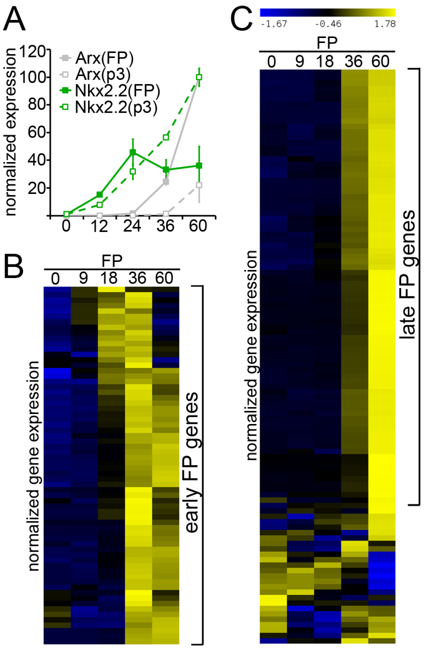

Figure S3. Identification of genes specific for definitive FP – "late" FP genes. Related to Figure 2 and Table S1, sheet S3.

A. The FP marker Arx is induced between 36 and 60h, at the time Nkx2.2 expression is downregulated.

B, C. Classification of FP genes. Genes expressed higher in iNPC-FP compared to iNPC-p3 at 36 and/or 60h were classified according to their time of induction as "early" FP genes, induced to maximum levels at 18 or 36h (B), or as "late" FP genes, induced to maximum levels at 60h (C).

Figure S4

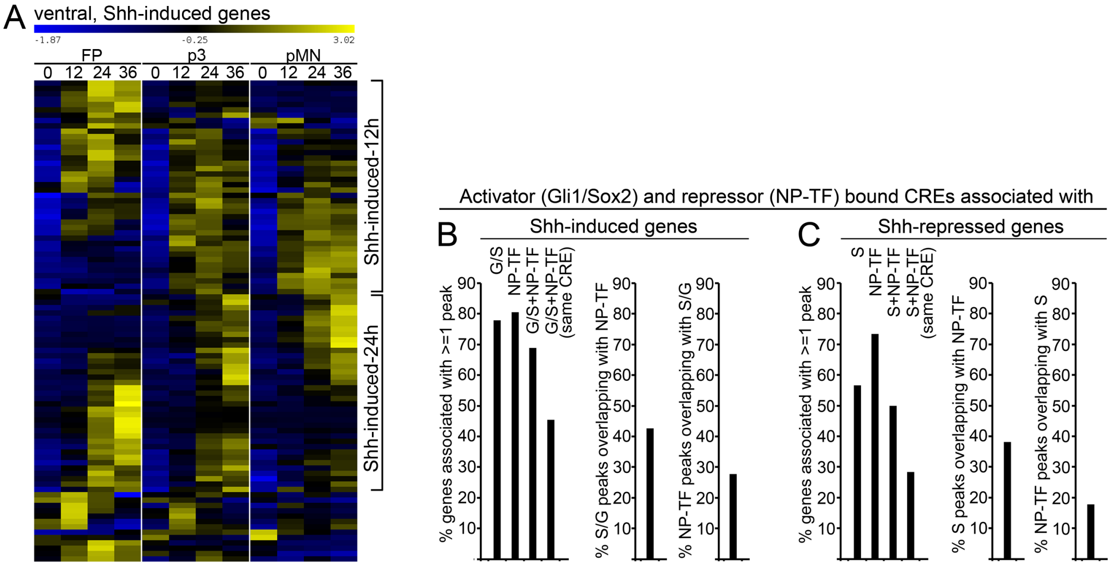

Figure S4. Expression kinetics of genes induced by Shh. Shh-regulated genes associate with activator and repressor peaks. Related to Figure 6 and Table S1, sheet S4.

A. Genes induced at 12h in FP, p3 or pMN were classified 'ventral-induced by Shh at 12h'. Genes induced at 24h in FP, p3 or pMN were classified 'ventral-induced by Shh at 24h'.

B-C. Most Shh-regulated genes associate with both activator and repressor peaks. Only a fraction of gene-associated CREs are co-occupied by activator and repressor TFs.

B. 78/81% of Shh-induced genes are associated with an activator (G/S, Gli1 and/or Sox2) or a repressor (NP-TF, Nkx2.2 or Olig2 or Nkx6.1), respectively. 88% of Sox2 and/or Gli1 associated genes bind a repressor as well (58% of those have at least one shared S/G+NP-TF CRE). 43% of activator bound peaks next to Shh-induced genes are bound by a repressor. 28% of repressor bound peaks are bound by an activator.

C. 57% of Shh-repressed genes are associated with an activator (S, Sox2), 73% with a repressor (NP-TF, Nkx2.2 or Olig2 or Nkx6.1). 88% of Sox2 associated genes bind a repressor as well (50% of those have at least one shared S+NP-TF CRE). 38% of activator bound peaks next to Shh-repressed genes are bound by a repressor. 18% of repressor bound peaks next to Shh-repressed genes is associated with an activator peak.

Figure S5

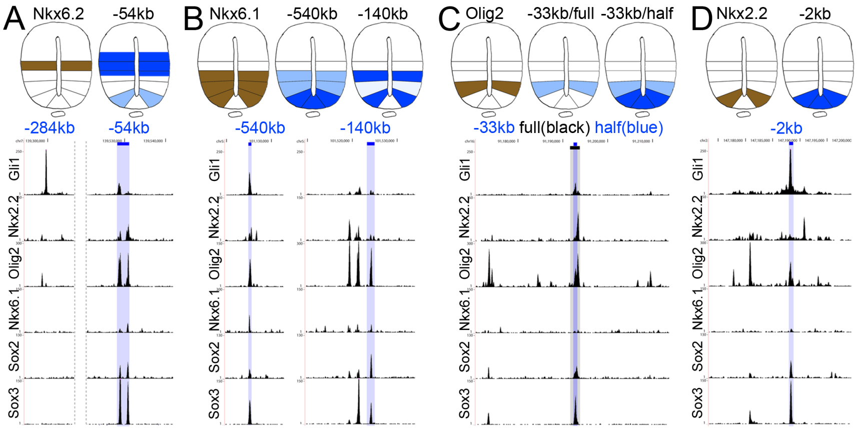

Figure S5. Activity of individual ventral CREs correlates with direct positive input of Sox2/3 and Gli1 and direct negative inputs of Nkx2.2, Olig2 and Nkx6.1. Related to Figures 5 and 6.

The schematics summarize dorsal-ventral restricted activities in the neural tube of the indicated CREs (blue, darker shades indicate higher expression) and compare them to domain-specific expression of the associated genes (brown). The tracks correspond to binding of Nkx2.2, Nkx6.1 and Sox2 in p3 and Olig2 in pMN cells. Gli1 tracks were generated by remapping the raw reads from (Peterson et al., 2012). Sox3 tracks were remapped from (Bergsland et al., 2011).

A. Nkx6.2(-54kb) double CRE which is strongly expressed in p2/p1-p0 domains and weakly in the p3 domain, is bound by Gli1, Sox TFs and Olig2 in pMN and by Nkx2.2 in p3 domains. CRE activity adapted from (Peterson et al., 2012).

B. Nkx6.1(-540kb) CRE which is strongly expressed in FP and p3 and weakly in pMN and p2, receives inputs from Gli1 and Sox TFs as well as from Olig2 and Nkx6.1. CRE activity adapted from (Peterson et al., 2012). Nkx6.1(-140kb) CRE expressed in p2 and p3 domains, receives positive inputs from Sox TFs and negative input from Olig2 in the pMN domain. CRE activity adapted from (Peterson et al., 2012).

C. The complete Olig2(-33kb) CRE is bound by Sox/Gli1 and by Olig2 and Nkx2.2. Its activity is detected in pMN and it is excluded from p3. The full CRE contains one Gli1 site, two Olig2 sites and an Nkx2.2 binding site. The truncated version of the same CRE, Olig2(-33kb/half), which lacks the Nkx2.2 element but retains the Sox, Gli and one of the Olig2 binding sites is active in the p3 domain consistent with a role for Nkx2.2 binding in the repression of this element in p3 domain the pMN domain. The Olig2 binding correlates with ability of Olig2 to repress its own expression (Fig. 5F). Full CRE activity adapted from (Oosterveen et al., 2012). Half CRE activity adopted from (Peterson et al., 2012).

D. p3-restricted activity of Nkx2.2(-2kb) CRE is consistent with the activatory inputs of Gli1/Sox binding and direct repression by Olig2 and more dorsal TFs (e.g Pax6, Irx3). The CRE activity was adapted from (Oosterveen et al., 2012; Peterson et al., 2012).

Supplemental Tables

Table S1. Genes, their expression and classification related to Figures 1D, 2B, 2F, 3B, 3F, 3G, S3B-C and S4A.

Sheet 1D: list of gene IDs, gene names, gene classification, SP-PIR-keywords, *in vivo* expression patterns, normalized expression values and p-values corresponding to the heatmap in Fig. 1D (dorsal-pMN-p3-FP progenitor genes). FP>p3>>pMN genes (Fig. 5B) and pMN>d genes (Fig. 5F) are indicated. The sheet 1D also contains the list of references corresponding to the *in vivo* gene expression patterns (bottom of the page).

Sheet 2B: list of gene IDs, gene names, gene classification, normalized expression values and p-values corresponding to the heatmap in Fig. 2B (pMN genes and pMN genes repressed by Nkx2.2).

Sheet 2F: list of gene IDs, gene names, SP-PIR-keywords, *in vivo* expression patterns, gene classification, normalized expression values and p-values corresponding to the heatmap in Fig. 2F (late FP genes and FP genes repressed by Nkx2.2). Sheet 2F also contains the list of references corresponding to the *in vivo* gene expression patterns (bottom of the page).

Sheet 3B: list of gene IDs, gene names, gene classification, normalized expression values and p-values corresponding to the heatmap in Fig. 3B (dorsal genes repressed in p3 by Nkx2.2 and/or Nkx6.1).

Sheet 3F: list of gene IDs, gene names, gene classification, normalized expression values and p-values corresponding to the heatmap in Fig. 3F (dorsal genes repressed in p3 and/or pMN).

Sheet 3G: list of gene IDs, gene names, gene classification, normalized expression values and p-values corresponding to the heatmap in Fig. 3G (dorsal genes repressed in pMN by Olig2).

Sheet S3: list of gene IDs, gene names, gene classification, normalized expression values and p-values corresponding to the heatmaps in Fig. S3B-C (early and late FP genes).

Sheet S4: list of gene IDs, gene names, gene classification, normalized expression values and p-values corresponding to the heatmaps in Fig. S4A (ventral genes activated by Shh at 12h and 24h).

Table S2. Lists of RNA and ChIP-seq samples, accession numbers. Related to Figures 1, 2, 3, 4, 5, 6, S1, S3, S4 and S5.

Sheet RNA-seq: list of conditions, corresponding RNA-seq samples and their ENA accession numbers.

Sheet ChIP-seq: list of ChIP-seq samples and their ENA accession numbers.

Table S3. RNA-seq: read counts. Related to Figures 1, 2, 3, 4, 5, 6, S1, S3 and S4.

Columns 1-140: Read counts - RNA-seq samples.

Columns 142-281: Read counts normalized - RNA-seq samples.

Table S4. ChIP-seq: peaks. Related to Figures 1, 2, 3, 5, 6, S1, S4 and S5.

Sheet Nkx2.2: position and read coverage of Nkx2.2 peaks (p3 cells).

Sheet Olig2: position and read coverage of Olig2 peaks (pMN cells).

Sheet Nkx6.1: position and read coverage of Nkx6.1 peaks (p3 cells).

Sheet Gli1: position and read coverage of Gli1 peaks (Peterson et al., 2012).

Sheet Sox2-d: position and read coverage of Sox2 peaks (dorsal cells).

Sheet Sox2-p3: position and read coverage of Sox2 peaks (p3 cells).

Sheet Genes-Peaks: List of RefSeq genes and associated peaks.

Table S5. Gene lists and samples used to generate heatmaps and boxplots. Related to Figures 1, 2, 3, 4, 5, 6, S1, S3 and S4.

The table contains multiple sheets, each sheet corresponds to the indicated figure panel.

Supplemental Experimental Procedures

Production of anti-Nkx2.2 and anti-Nkx6.1 antibodies

The N-terminal fragment of mouse Nkx2.2 (amino acids 26 to 121) was expressed as a His-tag fusion in E. *coli*. The C-terminal fragment of mouse Nkx6.1 (amino acids 300 to 365) was expressed as a GST-3C-Nkx6.1 fusion in E. coli. The GST was removed using HRV 3C protease (Millipore). Rabbits were immunized with purified proteins by Harlan, UK under licence PPL60/3418 following their predefined 77 day immunization schedule. The project was initiated following standard Ethical Review Process of Harlan Laboratories. The antibodies were affinity purified from the sera by coupling the immunogens to NHS-activated Sepharose (GE Healthcare).

Production of recombinant C25II-Shh protein

Mouse N-terminal fragment of Shh (amino acids 25-198, where the N-terminal cysteine 25 was replaced by two isoleucines) (Pepinsky et al., 1998) was cloned into pET22b downstream of a His-tag followed by enterokinase cleavage site, expressed and purified as described previously for unmodified human N-terminal Shh fragment (Williams et al., 1999).

Antibodies for immunohistochemistry

Following antibodies were used to stain chick sections and/or *in vitro* differentiated neural progenitor cells: Arx (rabbit, gift from J. Chelly (Poirier et al., 2004)), Dbx1 (rabbit, gift from T. Jessell (Vallstedt et al., 2001)), Foxa2 (rabbit, Seven Hills WRAB-1200 or goat, Santa Cruz sc-6554X), Irx3 (gift from T. Jessell (Novitch et al., 2001)), Nkx2.2 (mouse, DSHB 74.5A5 or rabbit, this manuscript), Olig2 (rabbit, Millipore AB9610), Nkx6.1 (mouse, F55A10 and F64A6B4, gift from O. Madsen, BCBC Antibody Core), Pax6 (rabbit, Millipore AB2237), Pax7 (DSHB).

ChIP-seq analysis

36-bp single-end reads were aligned to GRCm38 genome assembly using Bowtie (-v 0 -m 1 –best –strata) (Langmead et al., 2009). MACS14 (Zhang et al., 2008) was used to call peaks (-m 10, 200 -bw 100 –nolambda). Ten thousand peaks with the highest -10*log10(pvalue) from each dataset were selected for further analysis. Peak Splitter and the top four thousand peaks from each dataset were used to call subpeaks. Peak lists were combined and the number of reads per peak recalculated covering 200pb (250bp for Gli1) on each side of the peak summit, allowing no duplicate reads to map to the same peak. Peaks on chromosomes Y and M and peaks overlapping with the following regions: chr2:98,666,255-98,667,005, chr9:3,000,070-3,027,155, chr11:3,086,238-3,224,949, chr14:19,415,511-19,419,916, chr17:39,842,761-39,849,213 were eliminated from further analysis. These regions contain repetitive sequences where reads accumulate in a non-specific manner in all datasets analysed. Distribution of peak heights was approximated using log-normal distribution fit and peaks with height corresponding to cumulative p-value above 0.67 (Nkx2.2, Olig2, Nkx6.1, Sox2) or 0.9 (Gli1) were used for the analysis. This corresponds to 2644 Nkx2.2 peaks (minimum 62 reads), 3080 Olig2 peaks (minimum 188 reads), 2479 Nkx6.1 peaks (minimum 61 reads) and 860 Gli1 peaks (minimum 70 reads). Sox2 ChIP-seq was performed from p3 (2200 peaks, minimum 36 reads) and dorsal progenitors (2350 peaks, minimum 37 reads). For Fig. 6C and Fig. S4B-C, the highest peaks called from the two datasets which show 65% overlap were pooled and associated with the closest genes. For full peak lists including heights, see Table S4. Peaks were associated with the closest genes (-1, +1, within 1MB) using CisGenome (Ji et al., 2008) and gene reference table containing GRCm38 RefSeq genes (Table S4, sheet Genes-Peaks). For numbers of peaks associated with each gene, also see Table S4, sheet Genes-Peaks. De-novo motif search was performed using Homer (Heinz et al., 2010) and the sequence covering 100bp on each side from the peak summit. TomTom (Gupta et al., 2007) was used to search for similar motifs in known datasets.

RNA-seq analysis. Gene lists. Heatmaps. Boxplots.

The paired-end reads were aligned to GRCm38 genome using Tophat (default parameters) (Trapnell et al., 2009). The number of reads corresponding to all features in Ensembl Genes 77 GTF table were counted using HTSeq (Anders et al., 2015). See Table S3, columns 1-140 for raw read counts. The pairwise differential expression analysis was performed using DESeq (Anders and Huber, 2010) using raw read counts. Genes with very low counts across the whole dataset were removed prior to analysis (Table S3, column 141, “removed”). Otherwise, samples were normalized to the total number of reads (Table S3, columns 142-181). All cut-offs refer to the normalized values. MEV 4.0 (www.tm4.org) (Saeed et al., 2003) was used for hierarchical clustering and to generate expression heatmaps. “padj” refers to adjusted p-value (n-binomial) as defined by DESeq. “n.c” genes represent 600 RefSeq genes with minimal fold change across the RNAseq data set, the “n.c” genes are highlighted in (Table S4, sheet Genes-Peaks).

Bedtools (Quinlan and Hall, 2010), Samtools (Li et al., 2009) were used to interrogate, process and combine the ChIP-seq and RNA-seq datasets.

Samples and conditions used to generate heatmaps and boxplot panels are described below. For details of gene lists, see Table S5.

Fig. 1D – Table S5, sheet Fig1D

To classify genes differentially expressed in dorsal(30RA), p3, pMN and FP, gene lists 1D-1 (36h-dorsal(30RA) higher than 36h-p3), gene lists 1D-2 (36h-dorsal(300RA) higher than 36h-pMN), 1D-3 minus 1D-6 (36h-p3 higher than 36h-pMN), 1D-4 minus 1D-7 (pMN higher than p3) and 1D-4 (early FP higher than p3) were combined. To generate the heatmap, the average expression values from the replicate samples (1D-8) corresponding to 36h-dorsal(30RA), 36h-p3, 36h-pMN, 36h-FP conditions were normalized across the four conditions (z-scores). The genes were classified according their normalized expression values into Fig. 1D-"dorsal", Fig. 1D-"p3", Fig. 1D-"pMN", Fig. 1D-"FP" genes.

"Dorsal" genes are expressed higher in 36h-dorsal(30RA) than 36h-p3 and pMN and they are expressed in 36h-dorsal(30RA) or 36h-dorsal(30RA)/FP. "pMN" genes are expressed higher in 36h-pMN than 36h-p3 and expressed in 36h-pMN, 36h-pMN/dorsal or 36h-pMN/FP. "p3" genes are expressed higher in 36h-p3 than 36h-pMN and 36h-dorsal, they are expressed in 36h-p3 or 36h-p3/FP. For full list details, including p-values and the classification of the genes from the merged list corresponding to Fig. 1D, see Table S1, sheet 1D.

Fig. 2B – Table S5, sheet Fig2B

To identify pMN genes repressed by Nkx2.2, the gene list defined in Fig. 1D as "pMN" (expressed higher in 36h-pMN than 36h-p3 and expressed in 36h-pMN, 36h-FP and 36h-pMN or 36h-dorsal and 36h-pMN) was used.

To generate the heatmap, the average expression values from the replicate samples (2B-1) corresponding to 0h-pMN, 12h-pMN, 24h-pMN, 36h-pMN, 24h-pMN+Nkx2.2, 36h-pMN+Nkx2.2 samples were normalized across all conditions (z-scores). Genes were classified according to their time of induction into early, intermediate and late. padj was calculated from 2B-2-padj comparing expression values in 36h-pMN and 36h-pMN+Nkx2.2. pMN genes repressed by Nkx2.2 with padj<0.05 were classified "repressed by Nkx2.2". For full list details, including p-values and classification, see Table S1, sheet 2B.

Fig. 2F -Table S5, sheet Fig2F

To identify late FP genes repressed by Nkx2.2, the gene list defined as "late" FP genes in Fig. S3C (expressed higher in 60h-FP than 60h-p3 and induced to maximum levels at 60h) was used. To generate the heatmap, the average expression values from the replicate samples (2F-1) corresponding to 0h-FP, 9h-FP, 18h-FP, 36h-FP, 60h-FP, 18h-FP+Nkx2.2, 36h-FP+Nkx2.2, 60h-FP+Nkx2.2 were normalized across all conditions (z-scores). padj was calculated from 2F-2-padj60 comparing expression values in 60h-FP and 60h-FP+Nkx2.2. For full list details, including p-values, see Table S1, sheet 2F.

Fig. 3B – Table S5, sheet Fig3B

To identify dorsal genes repressed by Nkx2.2, Nkx6.1 and/or Nkx2.2+Nkx6.1, defined in Fig. 1D as "dorsal" (expressed higher in 36h-dorsal(30RA) than 36h-p3 and 36h-p3) was used. To generate the heatmap, the average expression values from the replicate samples corresponding to 36h-dorsal(30RA), 36h-p3, 36h-dorsal+Nkx2.2, 36h-dorsal+Nkx6.1, 36h-dorsal+Nkx2.2+Nkx6.1 conditions from the replicate samples (3B-1) were normalized across all conditions (z-scores). Genes were classified into "downregulated by Nkx2.2", "downregulated by Nkx6.1" and "downregulated by Nkx2.2 and Nkx6.1" based on the level of repression compared to 36h-dorsal(30RA). For full list details, including p-values and classification, see Table S1, sheet 3B.

Fig. 3F - Table S5, sheet Fig3F

To identify dorsal genes repressed in p3 and/or pMN, two lists were combined: 3F-1 (higher in 36h-dorsal(30RA) than 36h-p3) and 3F-2 (higher in 36h-dorsal(300RA) than 36h-pMN). Average values from the replicate samples in 36h-p3 and 36h-pMN were expressed as percent of 36h-dorsal(30RA) and 36h-dorsal(300RA) average values respectively, 36h-dorsal(30RA) and 36h-dorsal(300RA) average values were set to 100. To generate the heatmap, percent values corresponding to 36h-dorsal, 36h-p3 and 36h-pMN were then normalised across the three conditions. Genes were classified into "dorsal repressed in pMN", "dorsal repressed in p3" and "dorsal repressed in p3 and pMN" based on the level of repression compared to 36h-dorsal. For full list details, including classification, see Table S1, sheet 3F.

Fig. 3G - Table S5, sheet Fig3G

To identify 36-dorsal(300RA) genes repressed by Olig2, the gene list including genes expressed higher in dorsal(300RA) than pMN, defined as "repressed in pMN" in Fig. 3F was used. To generate the heatmap, the average expression values from the replicate samples (3G-1) corresponding to 36h-dorsal(300RA), 36h-pMN, 36h-dorsal(300RA)+Olig2 conditions were normalized across all conditions (z-scores). Genes were classified into "downregulated by Olig2" based on the level of repression compared to 36h-dorsal(300RA). For full details, including p-values and classification, see Table S1, sheet 3G.

Fig. 4B - Table S5, sheet Fig4B

To generate the boxplots corresponding to 36-dorsal and 36-pMN conditions when Nkx2.2, Nkx2.2 and Nkx6.1 or Nkx2.2HD-EnR were induced, "dorsal" genes (higher in 36h-dorsal(30RA) than 36h-p3 and pMN, expressed in 36h-dorsal(30RA) or 36h-dorsal(30RA)/FP), "pMN" genes (higher in 36h-pMN than 36h-p3, expressed in 36h-pMN, 36h-pMN/dorsal or 36h-pMN/FP) and "p3" (higher in 36h-p3 than 36h-pMN and 36h-dorsal, expressed in 36h-p3 or 36h-p3/FP) gene lists from Fig. 1D were used.

For the boxplot of 'dorsal' gene behaviour, the median expression values from the replicate samples (4B-1-d) corresponding to 36h-dorsal(30RA), 36h-p3, 36h-dorsal(30RA)+Nkx2.2, 36h-dorsal(30RA)+Nkx2.2+Nkx6.1 and 36h-dorsal(30RA)+Nkx2.2HD-EnR conditions, were normalized across all conditions (z-scores).

For the boxplot of 'pMN' gene behaviour, the median expression values from the replicate samples (4B-2-pMN) corresponding to 36h-pMN, 36h-p3, 36h-pMN+Nkx2.2 and 36h-pMN+Nkx2.2HD-EnR conditions, were normalized across all conditions (z-scores).

For 'p3' gene behaviour, the median expression values from the replicate samples (4B-3-p3) corresponding to 36h-dorsal(30RA), 36h-p3, 36h-dorsal(30RA)+Nkx2.2, 36h-dorsal(30RA)+Nkx2.2+Nkx6.1, 36h-dorsal(30RA)+Nkx2.2HD-EnR, 36h-pMN, 36h-pMN+Nkx2.2 and 36h-pMN+Nkx2.2HD-EnR conditions were normalized across all conditions (z-scores). Nkx2.2 read count in 36h-dorsal(30RA)+Nkx2.2, 36h-dorsal(30RA)+Nkx2.2+Nkx6.1 and 36h-dorsal(30RA)+Nkx2.2HD-EnR conditions was set to 600.

Fig. 4C - Table S5, sheet Fig4C

To generate the cross-correlation plots of dorsal/p3 and pMN/p3 gene behaviour when Nkx2.2, Nkx6.1, Nkx2.2 and Nkx6.1 or Nkx2.2HD-EnR were overexpressed, we used the same gene lists and same samples that were analysed in Fig. 4B.

For the cross-correlation of 'd-p3 genes', the median expression values from the replicate samples (4C-1-d-p3) corresponding to 36h-dorsal(30RA), 36h-p3, 36h-dorsal(30RA)+Nkx2.2, 36h-dorsal(30RA)+Nkx6.1, 36h-dorsal(30RA)+Nkx2.2+Nkx6.1, 36h-dorsal(30RA)+Nkx2.2HD-EnR conditions, were normalized across all conditions (z-scores).

For the cross-correlation of 'pMN-p3 genes', the median expression values from the replicate samples (4C-2-pMN-p3) corresponding to 36h-pMN, 36h-p3, 36h-pMN+Nkx2.2 and 36h-pMN+Nkx2.2HD-EnR conditions, were normalized across all conditions (z-scores). Nkx2.2 read count in 36h-dorsal(30RA)+Nkx2.2, 36h-dorsal(30RA)+Nkx2.2+Nkx6.1 and 36h-dorsal(30RA)+Nkx2.2HD-EnR conditions was set to 600.

Fig. 5B - Table S5, sheet Fig5B

To generate the boxplots corresponding to 36-FP, 36-FP+Nkx2,2, 36-p3, 36pMN+Nkx2.2, “FP>p3>>pMN” genes (“p3” genes expressed higher in 36h-FP than 36h-p3) from Fig. 5B, green arrow, were used. The average expression values from the replicate samples (5B-1) were normalized across all conditions (z-scores).

Fig. 5C - Table S5, sheet Fig5C

To identify “FP>p3>>pMN” genes downregulated by Nkx2.2, replicate samples corresponding to 36-FP and 36-FP+Nkx2.2 (list 5C-1) were compared.

Fig. 5F - Table S5, sheet Fig5F

To generate the boxplots corresponding to 24-dorsal, 24-dorsal+Olig2, 24-ventral, 24-ventral+Olig2, “pMN-only” genes (“pMN” genes expressed higher in 36h-pMN than 36h-p3 and 36h-dorsal) from Fig. 5F, red arrow, were used. The average expression values from the replicate samples (5F-1) were normalized across all conditions (z-scores). Olig2 values were adjusted according to qPCR data from the same RNA samples, using primers that do not amplify Olig2 cDNA.

Fig. 5G - Table S5, sheet Fig5G

To generate the boxplots corresponding to 24-dorsal, 24-ventral, 24-ventral+Olig2, “ventral” genes expressed in both p3 and pMN (list 5G-1) were used. The average expression values from the replicate samples (5G-1) were normalized across all conditions (z-scores).

Fig. 5H - Table S5, sheet Fig5H

To identify pMN-specific and ventral genes downregulated by Olig2, replicate samples corresponding to 24-p3 and 24-p3+Olig2 were compared (list 5H-1).

Fig. 6B - Table S5, sheet Fig6B

Ventral genes induced by Shh at 12h or 24h were defined as described in Fig. S4A.

To define dorsal genes repressed by Shh at 24h, two gene lists were combined: 6B-3-Shh-repressed (higher in 24h-dorsal(30RA) than 24h-p3) and 6B-4-Shh-repressed (higher in 24h-dorsal(300RA) than 24h-pMN).

Fig. 6C

Ventral genes (genes induced by Shh at 12h or 24h) and dorsal genes (genes repressed by Shh at 24h) are defined in paragraph Fig. 6B.

Fig. S3B,C – Table S5, sheet FigS3

To identify early and late FP genes, gene lists S3-1 (higher in 36h-FP than 36FP-p3) and S3-2 (higher in 60h-FP than 60FP-p3) were combined. To generate the heatmap, the average expression values from the replicate samples (S3-3) corresponding to 0h-FP, 9h-FP, 18h-FP, 36h-FP, 60h-FP conditions were normalized across all conditions (z-scores). FP genes were classified into “FP-early” (genes induced to maximum levels at 18 or 36h) and “FP-late” (genes induced to maximum levels at 60h) based on the kinetics of induction. For full list details, including classification, see Table S1, sheet S3B,C.

Fig. S4A - Table S5, sheet FigS4A.

To define genes induced by Shh at either 12h or 24h in ventral progenitors, two gene lists were combined: S4-1 (higher in 24h-p3 than 24h-dorsal(30RA)) and S4-2 (higher in 24h-pMN than 24h-dorsal(300RA)). To generate the heatmap, the average expression values from the replicate samples (S4-3) corresponding to 0h-FP, 12h-FP, 24h-FP, 36h-FP, 0h-p3, 12h-p3, 24h-p3, 36h-p3, 0h-pMN, 12h-pMN, 24h-pMN, 36h-pMN samples were normalized across all conditions (z-scores). Genes were classified according their time of induction in FP, p3 or pMN progenitors into “Shh-induced-12h” and “Shh-induced-24h”. For full list details, including classification, see Table S1, sheet S4.

Fig. S4B-C
Shh-induced genes (genes induced by Shh at 12h or 24h) and Shh-repressed genes (genes repressed by Shh at 24h) are defined in paragraph Fig. 6B.

Supplemental References

Berger, M.F., Badis, G., Gehrke, A.R., Talukder, S., Philippakis, A.A., Peña-Castillo, L., Alleyne, T.M., Mnaimneh, S., Botvinnik, O.B., Chan, E.T., et al. (2008). Variation in homeodomain DNA binding revealed by high-resolution analysis of sequence preferences. Cell *133*, 1266–1276.

Chen, C.Y., and Schwartz, R.J. (1995). Identification of novel DNA binding targets and regulatory domains of a murine tinman homeodomain factor, nkx-2.5. J. Biol. Chem. *270*, 15628–15633.

Huang, D.W., Sherman, B.T., and Lempicki, R.A. (2009). Systematic and integrative analysis of large gene lists using DAVID bioinformatics resources. Nature Protocols *4*, 44–57.

Jolma, A., Yan, J., Whitington, T., Toivonen, J., Nitta, K.R., Rastas, P., Morgunova, E., Enge, M., Taipale, M., Wei, G., et al. (2013). DNA-binding specificities of human transcription factors. Cell *152*, 327–339.

Li, H., Handsaker, B., Wysoker, A., Fennell, T., Ruan, J., Homer, N., Marth, G., Abecasis, G., Durbin, R. (2009). 1000 Genome Project Data Processing Subgroup. The Sequence Alignment/Map format and SAMtools. Bioinformatics *25*, 2078–2079.

Mazzoni, E.O., Mahony, S., Iacovino, M., Morrison, C.A., Mountoufaris, G., Closser, M., Whyte, W.A., Young, R.A., Kyba, M., Gifford, D.K., et al. (2011). Embryonic stem cell-based mapping of developmental transcriptional programs. Nat. Methods *8*, 1056–1058.

Pepinsky, R.B., Zeng, C., Wen, D., Rayhorn, P., Baker, D.P., Williams, K.P., Bixler, S.A., Ambrose, C.M., Garber, E.A., Miatkowski, K., et al. (1998). Identification of a palmitic acid-modified form of human Sonic hedgehog. J. Biol. Chem. *273*, 14037–14045.

Poirier, K., Van Esch, H., Friocourt, G., Saillour, Y., Bahi, N., Backer, S., Souil, E., Castelnau-Ptakhine, L., Beldjord, C., Francis, F., et al. (2004). Neuroanatomical distribution of ARX in brain and its localisation in GABAergic neurons. Brain Res. Mol. Brain Res. *122*, 35–46.

Quinlan, A.R., Hall, I.M. (2010). BEDTools: a flexible suite of utilities for comparing genomic features. Bioinformatics *26*, 841–842.

Saeed, A.I., Sharov, V., White. J., Li, J., Liang, W., Bhagabati, N., Braisted, J., Klapa, M., Currier, T., Thiagarajan, M., et al. (2003). TM4: a free, open-source system for microarray data management and analysis. BioTechniques *34*: 374–378.

Williams, K.P., Rayhorn, P., Chi-Rosso, G., Garber, E.A., Strauch, K.L., Horan, G.S., Reilly, J.O., Baker, D.P., Taylor, F.R., Koteliansky, V., et al. (1999). Functional antagonists of sonic hedgehog reveal the importance of the N terminus for activity. J. Cell. Sci. *112 (Pt 23)*, 4405–4414.
